# Ag_8_(PEt_3_)_6_[Ge_9_(Hyp)_2_]_4_: Insight into the Formation Mechanism of the Intermetalloid Cluster Ag_12_[Ge_9_(Hyp)_2_]_6_

**DOI:** 10.3390/molecules31162897

**Published:** 2026-08-20

**Authors:** Kevin Woern, Claudio Schrenk, Andreas Schnepf

**Affiliations:** Institute of Inorganic Chemistry, University of Tubingen, Auf der Morgenstelle 18, 72076 Tübingen, Germany; kevin.woern@uni-tuebingen.de (K.W.); claudio.schrenk@uni-tuebingen.de (C.S.)

**Keywords:** silver, germanium, cluster coordination, intermetalloid cluster

## Abstract

Recently we could demonstrate that the reaction of the bissilylated metalloid cluster K_2_[Ge_9_(Hyp)_2_] (Hyp = Si(SiMe_3_)_3_) with phosphine-stabilized silver chlorides gives the intermetalloid cluster Ag_12_[Ge_9_(Hyp)_2_]_6_, featuring a silver core of twelve silver atoms surrounded by six [Ge_9_(Hyp)_2_] units. Thereby, the silver atoms in the core appear to have an oxidation state of less than +I, indicating that a redox chemistry also takes place during the formation. We now present a possible precursor or intermediate to the intermetalloid cluster in this reaction system with the formula Ag_8_(PEt_3_)_6_[Ge_9_(Hyp)_2_]_4_. In this compound only four [Ge_9_(Hyp)_2_] units are arranged in a plane and connected via eight silver atoms. This compound seems to contain only silver cations, shedding further light on the complex solution and coordination chemistry of [Ge_9_(Hyp)_2_]^2−^ with transition metal cations. As the intermetalloid cluster Ag_12_[Ge_9_(Hyp)_2_]_6_ can be obtained from dissolved crystals of Ag_8_(PEt_3_)_6_[Ge_9_(Hyp)_2_]_4_, this indicated that the Ag_8_ compound might be an intermediate (kinetic product) on the way to the Ag_12_ compound (thermodynamic product).

## 1. Introduction

The chemistry of silylated nine-atomic metalloid germanium clusters is a broad field, since Schnepf synthesized the cluster [Ge_9_[(Hyp)_3_]^−^ (Hyp = -Si(SiMe_3_)_3_) in 2003 from a metastable GeBr solution [1]. Later Sevov et al. were able to synthesize the same cluster through the generally more accessible reaction of the Zintl phase K_4_Ge_9_ with HypCl [2]. From this point onwards a lot of follow up reactions with this silylated Ge_9_ cluster were performed to investigate its reactivity. So, it was shown that this cluster can form various coordination compounds with transition metals. A variety of transition metal salts have been observed to form compounds with this cluster, featuring the general formula [TM(Ge_9_(Hyp)_3_)_2_]^x^ (TM = Mn, Zn, Cd, Hg, x = 0; TM = Cu, Ag, Au, x = −1) [3,4,5]. With the reduction of Fe^2+^ to Fe^+^, [Ge_9_(Hyp)_3_]^−^ has been shown to form the cluster [Ge_18_(Hyp)_6_] through oxidative coupling [6]. It has also been observed to reduce Ni^2+^ and Co^2+^ to their respective monovalent cations. Thereby it has been demonstrated that Fe^+^ and Co^+^ are isolated separately, while the Ni^+^ cations are isolated as two different clusters in which one and two Ni^+^ units are coordinated by the triangular sides of the [Ge_9_[(Hyp)_3_]^−^ cluster [5]. These findings suggest that [Ge_9_[(Hyp)_3_]^−^ exhibits some notable redox reactivity towards transition metals in the groups 8 to 10. On the other hand, the reactivity of [Ge_9_[(Hyp)_3_]^−^ towards group 11 transition metals is limited to pure coordination. This is shown by the reaction of this cluster with phosphine, carbene and Cp* stabilized group 11 salts where the transition metal is η^3^ coordinated by the cluster [7,8,9,10]. Another interesting nine-atomic germanium cluster is the bissilylated cluster [Ge_9_[(Hyp)_2_]^2−^, which was synthesized by our working group in 2016 [11]. It is closely related to the trissilylated cluster as it can be synthesized through the same reaction, starting from K_4_Ge_9_, with the difference in using two equivalents of HypCl. [Ge_9_[(Hyp)_2_]^2−^ can be functionalized to give various [Ge_9_[(Hyp)_2_R]^−^ compounds [11,12,13,14]. [Ge_9_[(Hyp)_2_]^2−^ also exhibits some interesting redox reactivity. In the presence of a trivalent lanthanide salt or a tetravalent actinide salt, an oxidative coupling reaction occurs [15]. The bissilylated cluster [Ge_9_[(Hyp)_2_]^2−^ reacts in accordance with the known reactivity of the trissilylated cluster [Ge_9_[(Hyp)_3_]^−^ with phosphine-stabilized group 11 salts to cluster compounds where the transition metal is only coordinated by the cluster. However, dimerization is observed due to the more open cluster core of [Ge_9_[(Hyp)_2_]^2−^ [16]. In contrast to these results, we have shown recently that in the reaction of [Ge_9_[(Hyp)_2_]^2−^ with phosphine-stabilized silver salts with the common form (R_3_P)AgCl an intermetalloid cluster is formed with the composition Ag_12_[Ge_9_(Hyp)_2_]_6_ [17]. This cluster consists of a silver core that is surrounded by six [Ge_9_[(Hyp)_2_]^2−^ units. Within this intermetalloid cluster six of the eight silver atoms can be seen as Ag(0) atoms, showing that a type of redox chemistry takes place. In continuation of this work, we now present a cluster compound with the formula Ag_8_(PEt_3_)_6_[Ge_9_(Hyp)_2_]_4_ which might be seen as a precursor for the formation of the intermetalloid Ag_12_[Ge_9_(Hyp)_2_]_6_ cluster. This result additionally links the cluster Ag_12_[Ge_9_(Hyp)_2_]_6_ to the literature known chemistry of [Ge_9_[(Hyp)_2_]^2−^. The formation of larger complexes out of Ge_9_ units is also known in the literature for Ge_9_ clusters without ligands [18,19].

## 2. Results and Discussion

The new cluster Ag_8_(PEt_3_)_6_[Ge_9_(Hyp)_2_]_4_ (**1**) (Hyp = Si(SiMe_3_)_3_) is synthesized via the reaction of the bissilylated cluster K_2_[Ge_9_(Hyp)_2_] with (Et_3_P)AgCl in thf at −78 °C. Consequently, the starting materials are the same we used to obtain the intermetalloid cluster Ag_12_[Ge_9_(Hyp)_2_]_6_ although now in a 1 to 1 ratio. The reaction conditions are also the same with thf as solvent, a reaction temperature of −78 °C and the extraction with pentane. The key difference is the temperature used to crystallize the cluster. While crystals of Ag_12_[Ge_9_(Hyp)_2_]_6_ are formed at 50 °C, cluster **1** is obtained only if the pentane extract is stored at −30 °C. At that temperature single crystals of the title compound are obtained after 3 days as dark red thin plates. Whenever the extract is stored at higher temperatures, black diamond shaped crystals can be obtained which can be assigned to Ag_12_[Ge_9_(Hyp)_2_]_6_. This indicates that **1** might be the kinetic product of the reaction, while Ag_12_[Ge_9_(Hyp)_2_]_6_ is the thermodynamic product.

**Figure 1:**
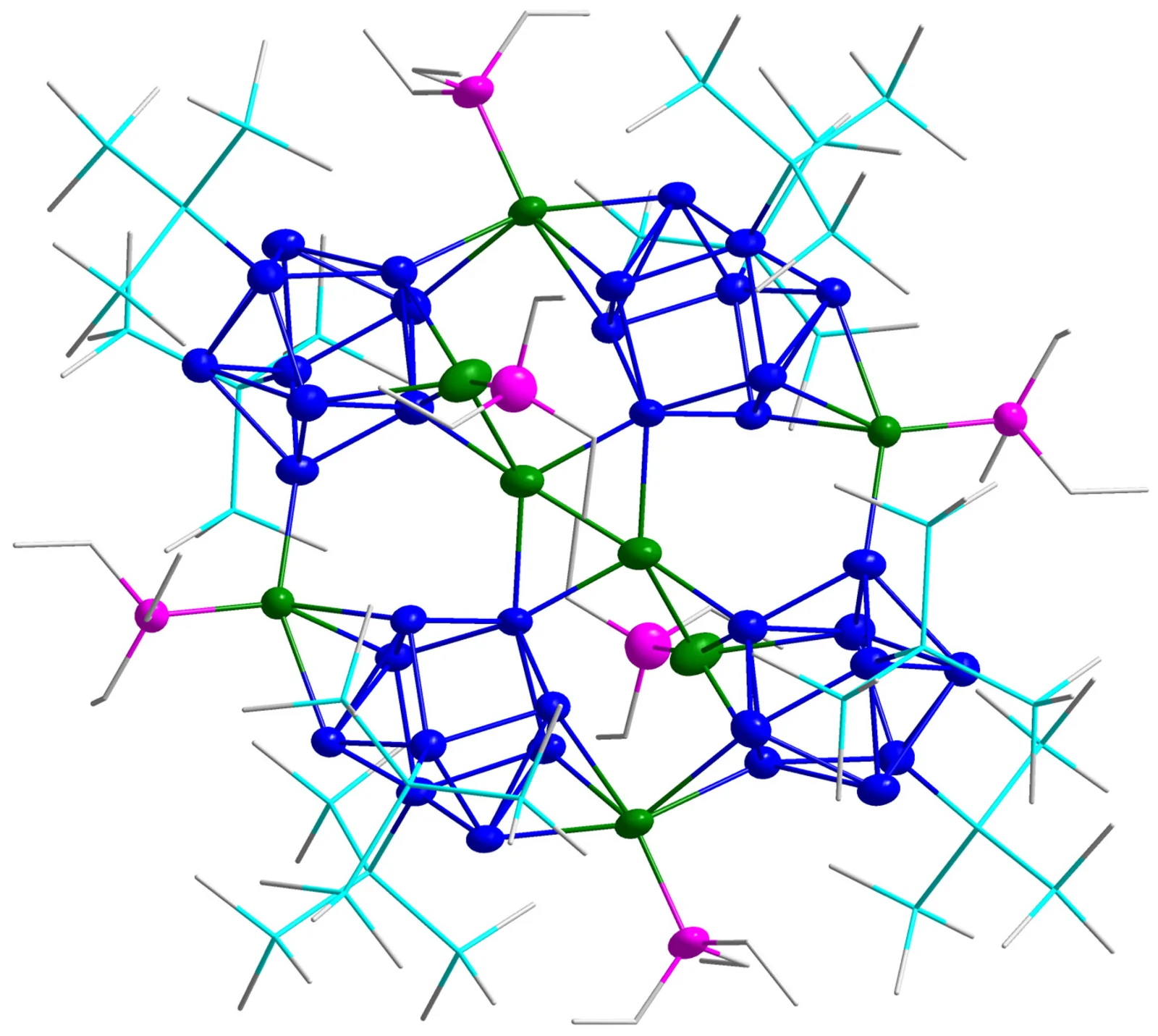
Molecular structure of **1**: Ag (green), Ge (blue) and P (pink) are shown as a displacement ellipsoid representation with 50% probability. Si (turquoise) and C (gray) are shown as wires/sticks. H atoms are omitted for clarity. (Selected bond lengths and angles are shown in the Table S2).

Crystals of **1** appeared as dark red thin plates, which is hardly surprising due to the fact that **1** can be seen as an oblate structure. These thin plates undergo layered growth. This leads to the fact that many of the crystals, which might be identified by the operator as a suitable single crystal candidate for x-ray diffraction, turned out to be, in most cases, a multi-domain crystal. However, after plenty of trials, we identified one suitable candidate for structure solution, where we observed a main domain of around 80% and this crystal was used to identify the molecular structure of **1** (Figure 1). The cluster compound **1** crystallizes in the triclinic space group P$\bar{1}$ with one molecule per unit cell (further details about structure refinement can be found in the Supporting Information).

Cluster **1** lays at the inversion center and therefore has C_i_ symmetry. It consists of four [Ge_9_(Hyp)_2_] units and eight silver atoms. Six of the eight silver atoms are stabilized by Et_3_P. The Ge_9_ units are all located in the same plane. They are arranged in that way that the Hyp ligands point out this plane at a 90° angle. Six silver atoms are in the same plane between the Ge_9_ units, connecting them to another. The remaining two silver atoms are above and below the plane bearing a phosphine ligand which is placed in the open space between the sterically demanding Hyp ligands (a space-filling model can be found in the Supplementary Materials, Figure S1).

**Figure 2:**
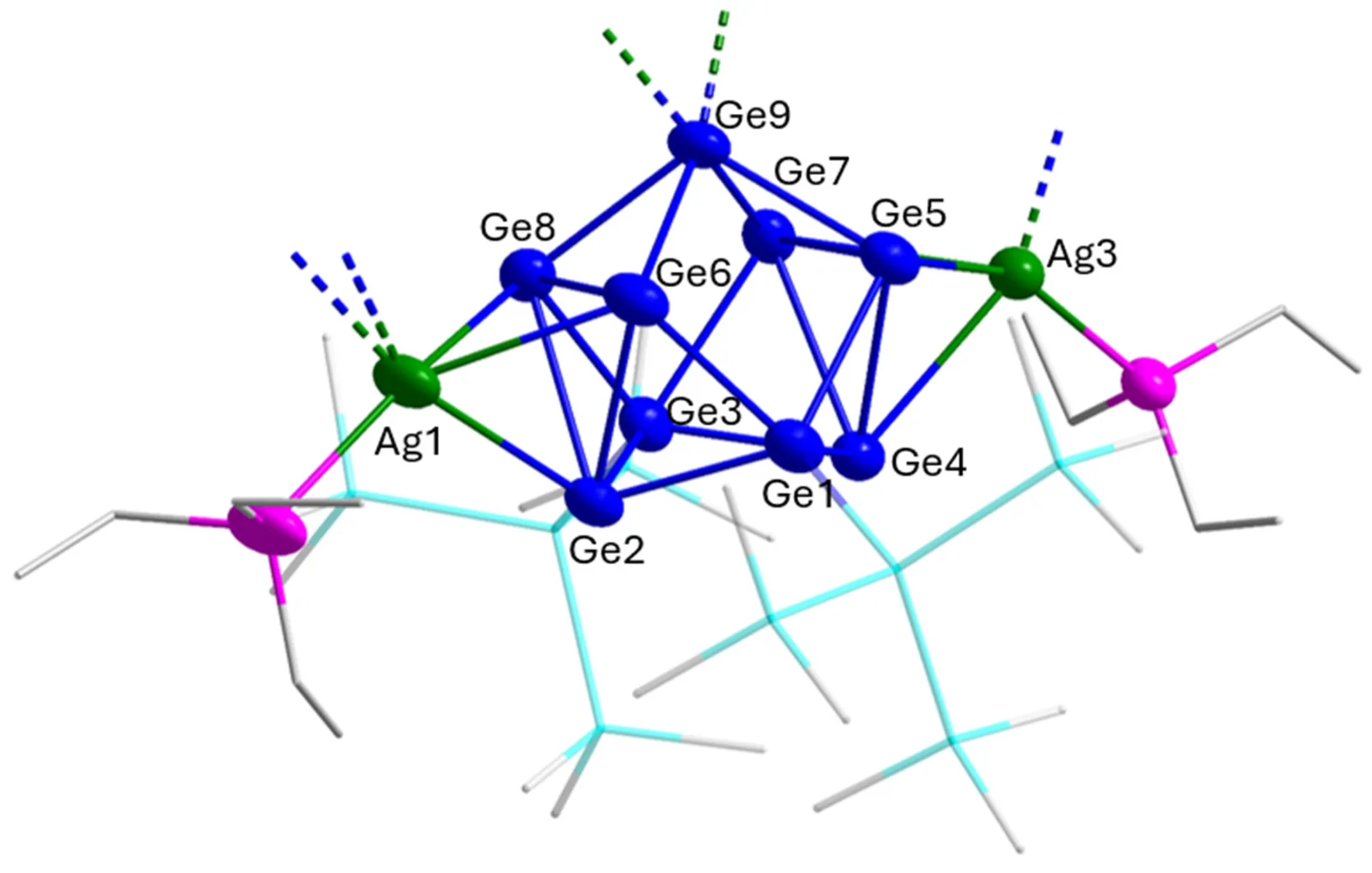
Molecular subunit [Ge_9_(Hyp)_2_(AgPEt_3_)_2_] **1a**. Ag (green), Ge (blue) and P (pink) are shown as a displacement ellipsoid representation with 50% probability. C (gray) and Si (turquoise) atoms are shown in the wires and stick model. Silicon bonds are transparent and H atoms are omitted for clarity. Further connections within **1** are indicated by dashed lines.

To better describe compound **1**, one can differentiate between two subunits in the molecular structure. Both subunits (**1a** and **1b**) consist of a [Ge_9_(Hyp)_2_] unit and two silver atoms. One subunit is a [Ge_9_(Hyp)_2_(AgPEt_3_)_2_] (**1a**) unit in which two phosphine-stabilized silver atoms are η^3^-coordinated to a [Ge_9_(Hyp_2_)] unit (Figure 2). Here, the Ge_9_ unit can be described as a distorted face capped trigonal prism (Figure S2 left). The silver atoms coordinate to the triangular faces of the prism. The Ag-Ge bonds in this subunit are in a range from 2.69 Å to 2.84 Å, which is in the same range as the Ag-Ge bonds in other known compounds featuring Ge_9_ and silver [4,9,17].

The structural motif found within subunit **1a** is already known in the literature. Thereby, the two best examples to which this structure can be compared are (NHC^Dipp^Cu_2_[Ge_9_(Hyp)_2_]) from the Fässler Group and the [(*n*Bu_3_P)AuGe_9_(Hyp)_2_Au(P*n*Bu_3_)]_2_ from our working group [16,20]. These two structures also show a bissilylated Ge_9_ cluster that coordinates with two coinage metal atoms, in these cases copper and gold respectively. The general arrangement of the atoms in these compounds is the same as in the substructure **1a**, with the coordination of the two phosphine-stabilized coinage metal atoms coordinated to the triangular faces of the trigonal prism. This subunit of the tetramer suggests that the first step of the reaction K_2_[Ge_9_(Hyp)_2_] with (Et_3_P)AgCl could be similar as in reactions involving the analogous copper and gold salts.

**Figure 3:**
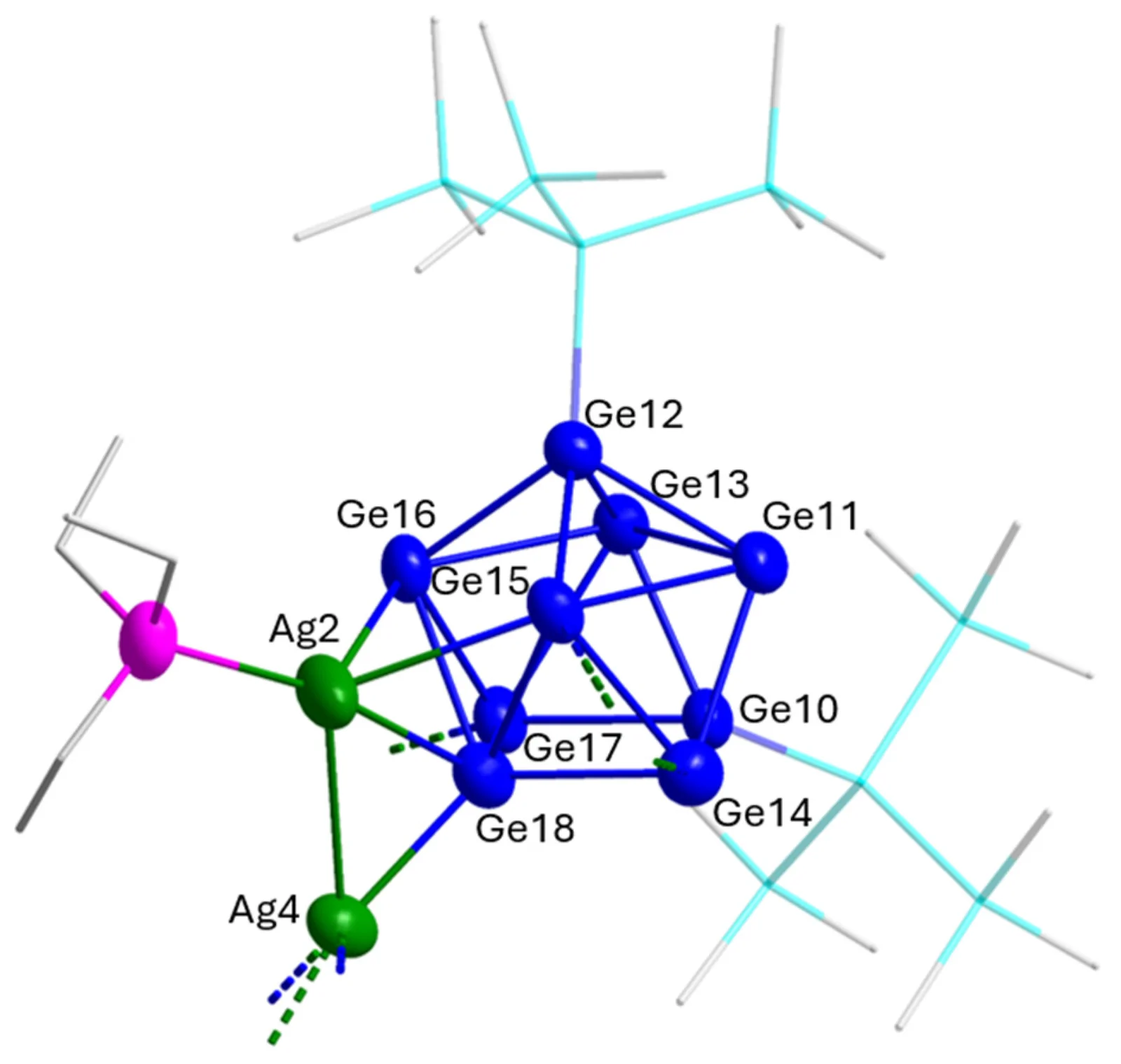
Molecular subunit [Ge_9_(Hyp)_2_(AgPEt_3_)Ag] **1b**. Ag (green), Ge (blue) and P (pink) are shown as a thermal ellipsoid representation with 50% probability. C (gray) and Si (turquoise) atoms are shown in the wires and stick model. Silicon bonds are transparent and H atoms are omitted for clarity. Further connections within **1** are indicated by dashed lines.

The second subunit, [Ge_9_(Hyp)_2_(AgPEt_3_)Ag] (**1b**), also contains a [Ge_9_(Hyp)_2_]^2–^ and two silver atoms. The first difference to **1a** is that there is only one silver atom stabilized by a phosphine (Figure 3). The second difference is that the Ge_9_ cluster in this subunit is distorted to the conformation of a mono-capped square antiprism (Figure S2 right). Compared to K_2_[Ge_9_(Hyp)_2_], the mono-capped square antiprism has a different orientation. Here, the square base is not spanned between the two germanium atoms bearing Hyp ligands, but between Ge10 bearing a Hyp ligand and Ge18, which is coordinated to two silver atoms (Figure 3). Ge12 which is bearing the second Hyp group serves as the capping atom of the conformation. The two silver atoms are, as mentioned, both coordinated to Ge18. The silver atom without a phosphine ligand is η^1^ coordinated while the second silver atom with phosphine ligand is η^3^ coordinated by the trigonal side of the cluster spanned between Ge18, Ge15 and Ge16. The Ge-Ge distance between Ge15 and Ge16 is elongated to 3.14 Å. The other Ge-Ge bond lengths of the upper square in the square antiprism spanned by Ge11, Ge13, Ge15 and Ge16 are also elongated to around 2.90 Å. These bond lengths are substantially larger than the other Ge-Ge bond lengths in the Ge_9_ unit which range from 2.49 to 2.71 Å. This elongation is in line with the literature-reported reactions of K_2_[Ge_9_(Hyp)_2_] with stabilized coinage metal halides [10,17]. The silver atoms in this subunit show a Ag-Ag-distance of 2.87 Å, which is slightly shorter than the bond length in elemental silver (2.89 Å) [21]. The Ge-Ag distances from Ge18 to the two silver atoms are 2.47 Å for the atom without phosphine and 2.58 Å for the silver atom with phosphine ligand. Both are shorter than the Ge-Ag distances in **1a** and more in the range of molecular Ge-Ag distances [22,23]. The silver atom with phosphine ligand is as mentioned coordinated by Ge15 and Ge16; the distances to those atoms are 2.81 Å which is in the range of other known Ge-Ag distances in Ge_9_-Ag chemistry and 3.00 Å which is substantially longer.

The subunits are connected to each other via the silver atoms. The Ge_9_ units of the two subunits **1a** are coordinating to the two silver atoms without a phosphine ligand. In this only the third capping germanium atom of the trigonal prism, Ge9, coordinates to both silver atoms. The Ge-Ag distances are 2.62 Å and 2.65 Å, in good accordance with Ge-Ag distances found in Ag_12_[Ge_9_(Hyp)_2_]_6_, here formed from the third capping germanium atom of the trigonal prism to two silver atoms of the inner tetrahedron of Ag_12_ [17]. The Ge_9_ cores of the subunit **1b** connect to the silver atoms of subunit **1a** through η^1^- and η^2^-coordination. Thereby, Ge17 coordinates via η^1^-coordination with a distance of 2.62 Å while Ge14 and Ge15 coordinate via η^2^-coordination with the distances 3.01 Å and 2.92 Å. The last contact in this cluster is the one between the two silver atoms without phosphine ligand. The two atoms have a contact length of 2.56 Å which is extremely short for an Ag-Ag contact. Contacts this short are known in the literature as for example the Ag_2_[PhC(NSiMe_3_)_2_]_2_ complex from Fenske et al. [24]. Another example is the rad shaped Supercluster Ag_61_(dpa)_27_(SbF_6_)_4_ (Hdpa = dipyridylamine) [25]. For a contact this short there is a possibility of a Ag-Ag bond [26]. To investigate this further we performed DFT calculations on the model compound Ag_8_(PH_3_)_6_[Ge_9_(Si(SiH_3_))_2_]_4_ to check whether we can find any hints of a covalent Ag-Ag-bond. First of all, we checked the calculated molecular orbitals. Since we used a model compound with simplified ligands, it should be easier to find an Ag-Ag bonding orbital. Down to HOMO-40 no such orbital was found. Most of the high level occupied orbitals were located at the four Ge_9_ units. This hints to an ionic situation with Ag formally charged as 1+, as expected. Secondly, we performed an Ahlrichs–Heinzmann population analysis to achieve the Shared Electron Numbers (SENs), which were stated out as 2-center, 3-center and 4-center contributions. The shared electron numbers (SEN) is a reliable value to describe covalent bonds [27]. We found a 2-center-SEN involving the two central Ag atoms of 0.52, which is significantly reduced compared to classical single bonds like in the Ag_2_ dimer [28], where an SEN of 0.919 is calculated. Looking at the 3-center contribution, we found an increased SEN of 0.24, where the two Ag atoms and the top Ge atom of the bridging Ge_9_ cluster are involved. This is not surprising if we state a bridging interaction of the Ge_9_ unit with two Ag^+^ cations. Since the HOMO of heavier congener [Sn_9_(Hyp)_2_]^2−^ was calculated as the p-symmetrical lone pair of the topping Sn atom [29], this result could be directly transferred to the isostructural Ge_9_ unit. So, the Ge_9_ unit can indeed provide electrons to bridge two cations. Since **1** (as well as the calculated model compound) is inversion-symmetric, this 3-center SEN is found two times. A 4-center contribution involving both Ge_9_ units and the two Ag atoms could not be identified. These results underline the argument that there is no covalent bond between the two central Ag atoms, even if a short Ag-Ag distance is realized. It is better described as two Ag^+^ cations glued together with the electron-rich [Ge_9_(Hyp)_2_]^2−^ unit.

As mentioned in the beginning, cluster compound **1** is not isolable at higher temperatures. Whenever the extract is stored at room temperature, black diamond-shaped crystals are formed which can be assigned to Ag_12_[Ge_9_(Hyp)_2_]_6_. Furthermore, we were able to obtain crystals of Ag_12_[Ge_9_(Hyp)_2_]_6_ from crystals of **1** dissolved in thf or benzene and stored at room temperature. This is a strong argument towards our assumption that **1** is a precursor for the intermetalloid cluster Ag_12_[Ge_9_(Hyp)_2_]_6_. The ^1^H-NMR spectrum suggests that **1** first decomposed in solution as one very broad signal at 0.35 ppm to 0.45 ppm can be seen. In this region is another broad signal at around 0.37 ppm. There are also two small distinct signals at 0.24 and 0.26 ppm (Figures S3 and S4). One likely decomposition product could be subunit **1a** as it is already known in the literature for the other phosphine-stabilized coinage metals. Following this assumption **1b** could also be another plausible decomposition product. We cannot for certain assign the subunits to the observed ^1^H-NMR signals. From these decomposition product’s further reaction steps take place until the cluster Ag_12_[Ge_9_(Hyp)_2_]_6_ is formed. Thereby, the phosphine ligands coordinated to the silver cations might be substituted by the coordination to germanium atoms of the Ge_9_(Hyp)_2_ units, leading to a complex equilibrium mixture in solution. From this mixture at the end the least soluble Ag_12_[Ge_9_(Hyp)_2_]_6_ I crystalizes from the solution.

## 3. Materials and Methods

All handling of materials was performed under exclusion of air and moisture, either by using dried Schlenk type flasks or an argon filled glovebox. Solvents were refluxed over drying agents (thf over sodium and pentane over CaH_2_) for one hour, then distilled and degassed before storage over a Na/K alloy. K_2_[Ge_9_(Hyp)_2_] was synthesized through a synthesis known from the literature [11]. (Et_3_P)AgCl was synthesized through the reaction of AgCl and Et_3_P in toluene.

Synthesis of Ag_8_(PEt_3_)_6_[Ge_9_(Hyp)_2_]_4_ (**1**): K_2_[Ge_9_(Hyp)_2_] (123 mg, 0.1 mmol, 1 eq.) and (Et_3_P)AgCl (26 mg, 0.1 mmol, 1 eq.) are put together and dissolved in thf (20 mL) at −78 °C. The brown solution is then stirred for an hour at −78 °C. In this hour the solution turns red. Afterwards thf is removed under reduced pressure. The dried reaction mixture is then extracted with pentane (10 mL). The deep red extract is afterwards stored at −30 °C for 36 h, where dark red crystals of **1** are formed as thin plates (Yield 2 mg, 0.03% in relation to K_2_Ge_9_(Hyp)_2_).

NMR-spectra were measured using a Burker AVIIIHD-300 spectrometer with a 4 mm H-1,X CP/MAS probe head. The chemical shifts are given in ppm against the external standard SiMe_4_. C_6_D_6_ was dried with 3 Å molecular sieve and thf-d8 was dried over a Na/K alloy.

EDX analysis was performed with a Bruker QUANTAX 6G, Typ 200, XFlash 6/60-250, SDD slew 60. (Bruker Nano GmBH, Berlin, Germany).

Single crystals of **1** were mounted on a Rigaku XtaLAB Synergy-S X-ray diffractometer at 150 K. The diffractometer is equipped with a PhotonJet-S source for monochromated CuKα radiation (λ = 1.54184 Å) and an Oxford Cryosystems cryostat. A semiempirical absorption correction using spherical harmonics was applied using the SCALE3 ABSPACK algorithm. The structure was solved by direct methods and refined against F^2^ for all observed reflections. Programs used: SHELXS and SHELXL within the Olex2 program package [30,31,32].

SADI, SIMU, ISOR and DFIX restraints were used to get a reasonable description of Si(SIMe_3_)_3_ groups. Due to non-refineable solvent molecules the routine SQUEEZE implemented in the PLATON program package [33] was used to identify two voids (226 and 227 Å^3^) in the unit cell containing 47 electrons each. This fits to one pentane molecule per void.

Quantum-chemical calculations were carried out with the RI-DFT version of the Turbomole program package by employing the BP86-functional. The basis sets were of SVP quality. The TmoleX client was used as graphical user interface and for illustrating the molecular orbitals [34,35,36,37,38,39,40,41,42,43].

## 4. Conclusions

We were able to get further insight into the reaction of K_2_[Ge_9_(Hyp)_2_] with a phosphine-stabilized silver chloride. In prior research we were able to synthesize the intermetalloid cluster Ag_12_[Ge_9_(Hyp)_2_]_6_. We now were able to isolate another cluster compound Ag_8_(PEt_3_)_6_[Ge_9_(Hyp)_2_]_4_ (**1**) from this reaction system, which seems to be a precursor for Ag_12_[Ge_9_(Hyp)_2_]_6_. Compound **1** is synthesized from the same educts but with a ratio of one to one instead of one to two and the temperature is always held at −30 °C. **1** can be described fully as a pure coordination compound. A substructure of **1** is already known in the literature. This connects the reactivity of K_2_[Ge_9_(Hyp)_2_] with phosphine-stabilized silver chlorides with the reactivity of K_2_[Ge_9_(Hyp)_2_] with equivalent copper and gold chlorides. Although in **1** a very short Ag-Ag distance is found for one Ag-Ag contact, DFT calculations show no indication of a covalent Ag-Ag bond. Finally, we were able to show that cluster **1** can be converted to Ag_12_[Ge_9_(Hyp)_2_]_6_, further supporting that **1** is a precursor to the larger intermetalloid cluster Ag_12_[Ge_9_(Hyp)_2_]_6_.

## Data Availability

The original contributions presented in this study are included in the article/Supplementary Materials. Further inquiries can be directed to the corresponding author.

## Supplementary Materials

The following supporting information can be downloaded at: https://www.mdpi.com/article/10.3390/molecules31162897/s1, Figure S1: Space-filling model of compound **1**; Figure S2: Molecular structure of subunit 1a (left) and subunit 1b (right): View to see the structural motive of the Ge_9_ cores; Figure S3: ^1^H-NMR Spectrum of the crystals of compound **1**; Figure S4: ^1^H-NMR Spectrum of the crystals of compound **1** in the region of 0 to 0.16 ppm; Figure S5: SEM Image of the crystals of compound **1**; Figure S6: EDX Spectra of measurement Points 2, 3 and 4; Figure S7: Geometry optimized structure; Figure S8: Visual presentation of the 2c-SEN; Table S1: Data of the crystal structure determination of compound **1**; Table S2: Selected Bond lengths for 1; Table S3: EDX measurement Point 2; Table S4: EDX measurement Point 3; Table S5: EDX measurement Point 4; Table S6: Atomic coordinates; Table S7: 2c-SEN; Table S8: 3c-SEN and 4c-SEN.

## Abbreviations

The following abbreviations are used in this manuscript:

Bu	Butyl
Cp*	Pentamethylcyclopentadien
DFT	Density function theory
Ri-DFT	Resolution of identity density functional theory
EDX	Energy-dispersive X-ray spectroscopy
Et	Ethyl
et al.	And others
HOMO	Highest occupied molecular orbital
Hyp	Tris(trimethylsilyl)silyl [-Si(SiMe_3_)_3_]
Hdpa	Dipyridylamine
LUMO	Lowest unoccupied molecular orbital
Me	Methyl
NHC	N-heterocylclic carbene
NHC^Dipp^	1,3 (2,6-diisopropylphenyl)imidazole-2-ylidene
NMR	Nuclear magnetic resonance
Ph	Phenyl
ppm	Parts per million
Pr	Propyl
XRD	X-ray diffraction
SEN	Shared electron number
2c-SEN	2-center shared electron number
3c-SEN	3-center shared electron number
4c-SEN	4-center shared electron number
SEM	Scanning electron microscopy
SVP	Split valence polarized
thf	Tetrahydrofuran
