# Supplementary material for: Ag8(PEt3)6[Ge9(Hyp)2]4: Insight into the Formation Mechanism of the Intermetalloid Cluster Ag12[Ge9(Hyp)2]6
Source: Molecules. 2026 Aug 20;31(16):2897. doi: 10.3390/molecules31162897 (PMC13515316; doi:10.3390/molecules31162897)
Supplement: Supplementary file 1 [file molecules-31-02897-s001.zip › molecules-4482696-supplementary.pdf]

Supporting Information

$Ag_8(PEt_3)_6[Ge_9(Hyp)_2]_4$:

Insight into the formation mechanism of the intermetalloid Cluster $Ag_{12}[Ge_9(Hyp)_2]_6$.

Kevin Woern, Claudio Schrenk and Andreas Schnepf *

Institut für Anorganische Chemie, Universität Tübingen, Auf der Morgenstelle 18,

72076 Tübingen, Germany

* Correspondence: andreas.schnepf@uni-tuebingen.de

1. Crystallographic Data

Table S1: Data of the crystal structure determination of compound **1**

Empirical formula	$C_{108}H_{306}Ag_8Ge_{36}P_6Si_{32}$
Formula weight	6166.41
Temperature/K	150.00(11)
Crystal system	triclinic
Space group	P-1
a/Å	15.94884(19)
b/Å	20.6136(3)
c/Å	20.8395(2)
α/°	71.2370(11)
β/°	78.1243(9)
γ/°	70.2873(11)
Volume/$Å^3$	6070.51(14)
Z	1
$\rho_{calc}g/cm^3$	1.687
μ/mm^{-1}	12.003
F(000)	3020.0
Crystal size/mm^3	0.349 × 0.134 × 0.089
Radiation	Cu Kα (λ = 1.54184)
2Θ range for data collection/°	4.506 to 128.752
Index ranges	-18 ≤ h ≤ 18, -19 ≤ k ≤ 24, -24 ≤ l ≤ 24
Reflections collected	86385
Independent reflections	20259 [Rint = 0.0541, Rsigma = 0.0345]
Data/restraints/parameters	20259/343/956
Goodness-of-fit on F^2	1.065
Final R indexes [I>=2σ (I)]	R1 = 0.0668, wR2 = 0.1823
Final R indexes [all data]	R1 = 0.0736, wR2 = 0.1890
Largest diff. peak/hole / e $Å^{-3}$	4.41/-2.17
CCDC number	2573842

Squeeze results:

Due to non-refineable solvent molecules the routine SQUEEZE implemented in the PLATON program package[1] was used to identify two voids (226 and 227 Å³) in the unit cell containing 47 electrons each. This fits to one pentane molecule per void.

Table S2: Selected Bond lengths for **1**

Atom	Atom	Length Å		Atom	Atom	Length Å
Ag1	Ge4	2.7911(12)		Ge3	Ge8	2.5475(15)
Ag1	Ge5	2.7842(12)		Ge3	Si1	2.386(2)
Ag1	Ge8	2.7904(13)		Ge4	Ge5	2.8476(13)
Ag1	Ge14	3.0751(14)		Ge4	Ge8	2.8013(14)
Ag1	Ge15	2.9166(13)		Ge5	Ge8	2.7543(15)
Ag1	P1	2.461(3)		Ge5	Ge9	2.5647(15)
Ag2	Ag4	2.8668(11)		Ge6	Ge7	2.7572(13)
Ag2	Ge15	2.8137(13)		Ge6	Ge9	2.5791(15)
Ag2	Ge16	3.0070(15)		Ge7	Ge9	2.5305(14)
Ag2	Ge18	2.5803(14)		Ge8	Ge9	2.5468(13)
Ag2	P2	2.402(3)		Ge10	Ge11	2.5343(14)
Ag3	Ge2[1]	2.8368(11)		Ge10	Ge13	2.5625(15)
Ag3	Ge6[1]	2.8444(11)		Ge10	Ge14	2.5568(15)
Ag3	Ge7[1]	2.6903(11)		Ge10	Ge17	2.5121(14)
Ag3	Ge17	2.6303(11)		Ge10	Si4	2.376(3)
Ag3	P3	2.461(2)		Ge11	Ge12	2.5274(15)
Ag4	Ag4[1]	2.5670(15)		Ge11	Ge13	2.8881(15)
Ag4	Ge9	2.6547(13)		Ge11	Ge14	2.6884(16)
Ag4	Ge9[1]	2.6202(11)		Ge12	Ge13	2.5227(14)
Ag4	Ge18	2.4735(14)		Ge12	Ge15	2.5233(15)
Ge1	Ge2	2.5280(13)		Ge12	Ge16	2.5239(14)
Ge1	Ge4	2.5046(13)		Ge12	Si3	2.385(3)
Ge1	Ge5	2.5431(14)		Ge13	Ge16	2.9133(14)
Ge1	Ge6	2.5570(12)		Ge13	Ge17	2.6626(14)
Ge1	Si2	2.385(2)		Ge14	Ge15	2.5867(17)
Ge2	Ge3	2.5301(14)		Ge14	Ge18	2.5308(16)
Ge2	Ge6	2.8127(13)		Ge15	Ge18	2.6590(15)
Ge2	Ge7	2.7667(13)		Ge16	Ge17	2.5348(16)
Ge3	Ge4	2.5072(13)		Ge16	Ge18	2.7151(17)
Ge3	Ge7	2.5448(12)		Ge17	Ge18	2.4894(16)

[1] 1-X,-Y,1-Z

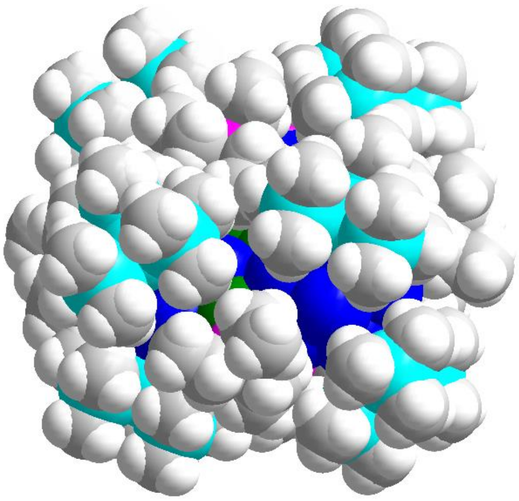

Figure S1: Space-filling model of compound **1**.

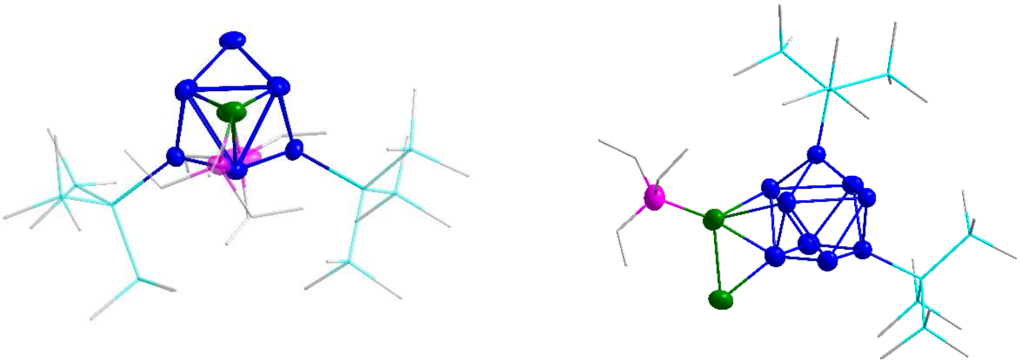

Figure S2: Molecular structure of subunit **1a** (left) and subunit **1b** (right): View to see the structural motive of the Ge_9 cores.

NMR Spectroscopy

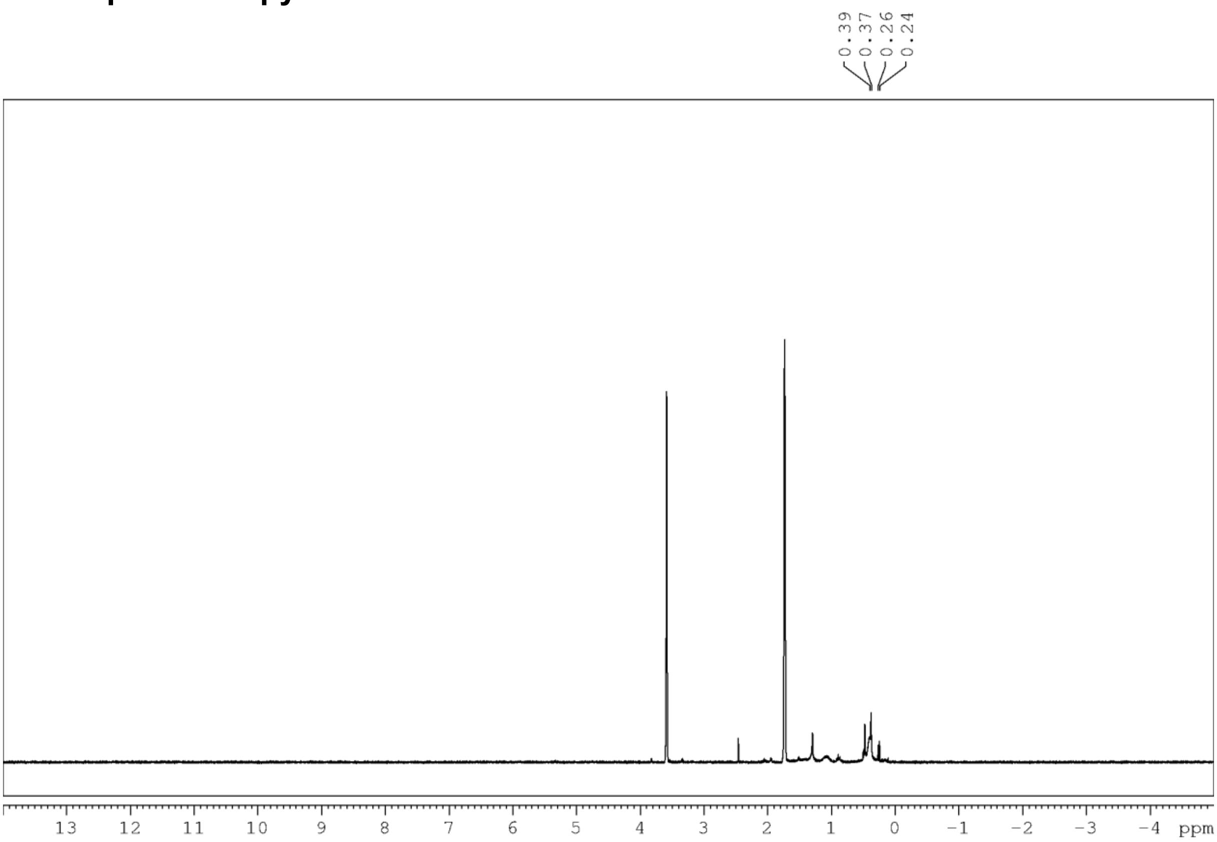

Figure S3: ^{1}H-NMR Spectrum of the crystals of compound **1**.

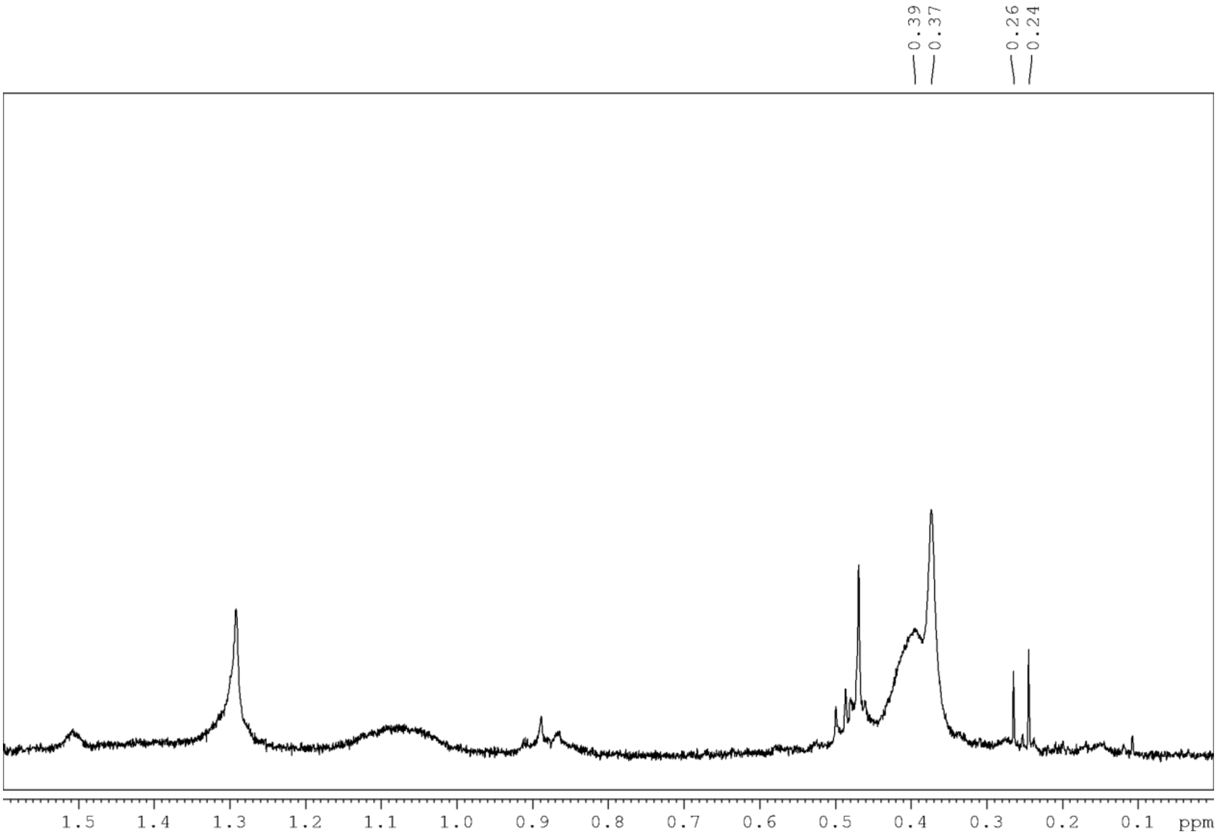

Figure S4: ^{1}H-NMR Spectrum of the crystals of compound **1** in the region of 0 to 1.6 ppm.

2. EDX Measurement Data

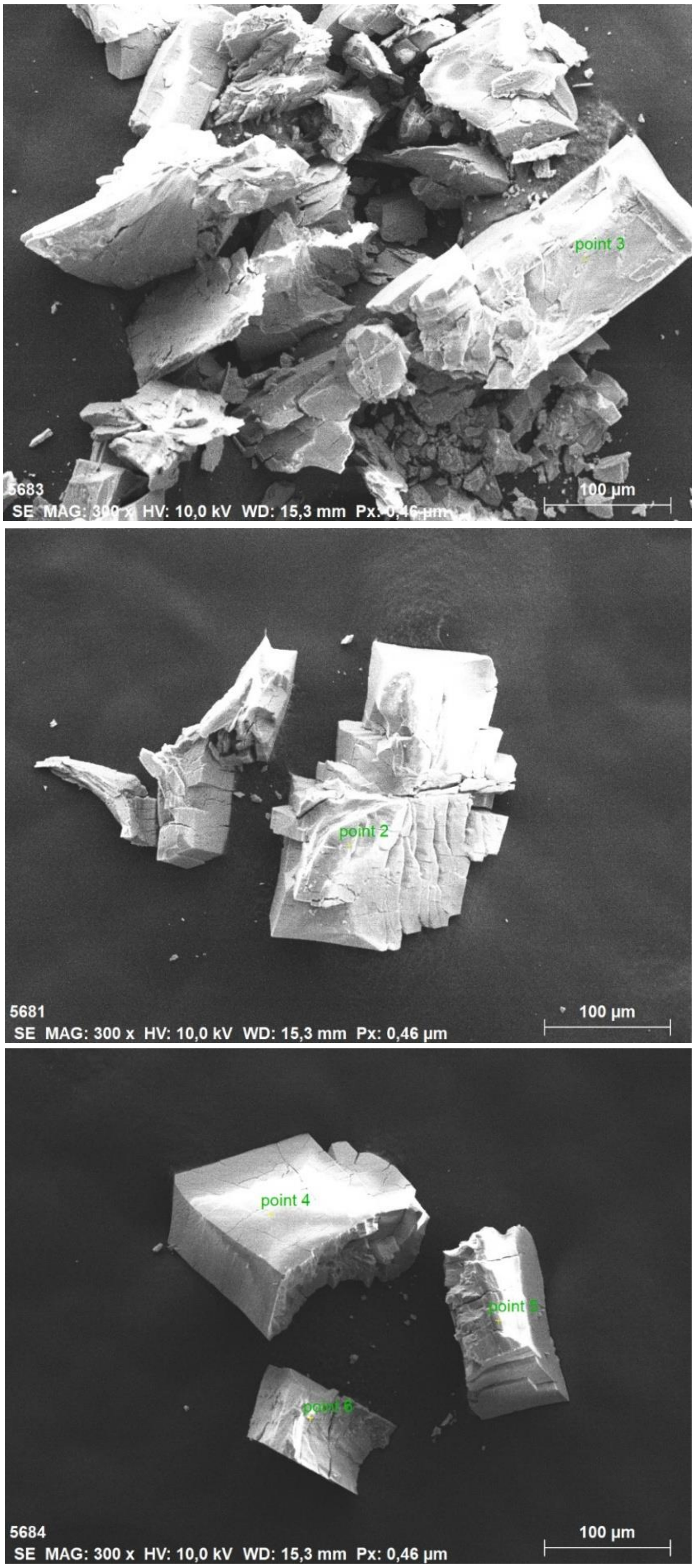

Figure S5: SEM Image of the crystals of compound **1**; EDX Measurement points are marked in green**.**

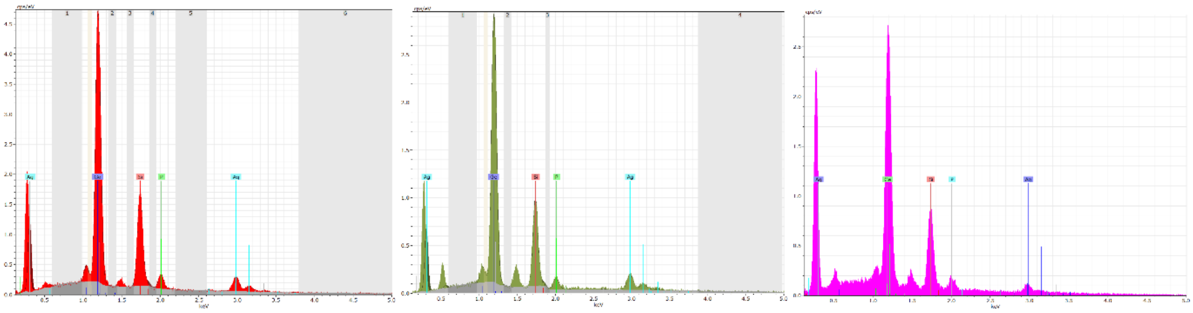

Figure S6: EDX Spectra of measurement Points 2, 3 and 4.

Table S3: EDX measurement Point 2

Element	Norm Wt. %	Narm Wt. % calculated	Norm At%	Norm At% calculated	Error (Wt% 3 σ)
Ge	56,56	57,23	42,54	43,90	6,47
Si	21,08	19,67	40,99	39,02	1,98
Ag	18,27	18,89	9,25	9,76	1,59
P	4,10	4,21	7,22	7,32	0,52

Table S4: EDX measurement Point 3

Element	Norm Wt. %	Narm Wt. % calculated	Norm At%	Norm At% calculated	Error (Wt% 3 σ)
Ge	56,83	57,23	42,84	43,90	5,68
Si	20,92	19,67	40,77	39,02	1,69
Ag	18,20	18,89	9,24	9,76	1,34
P	4,05	4,21	7,16	7,32	0,41

Table S5: EDX measurement Point 4

Element	Norm Wt. %	Narm Wt. % calculated	Norm At%	Norm At% calculated	Error (Wt% 3 σ)
Ge	56,60	57,23	42,72	43,90	4,21
Si	20,77	19,67	40,53	39,02	1,30
Ag	18,47	18,89	9,39	9,76	1,19
P	4,16	4,21	7,37	7,32	0,37

3. Quantum chemical calculations

model compound $Ag_8[Ge_9(Si(SiH_3)_3)_2]_4(PH_3)_6$

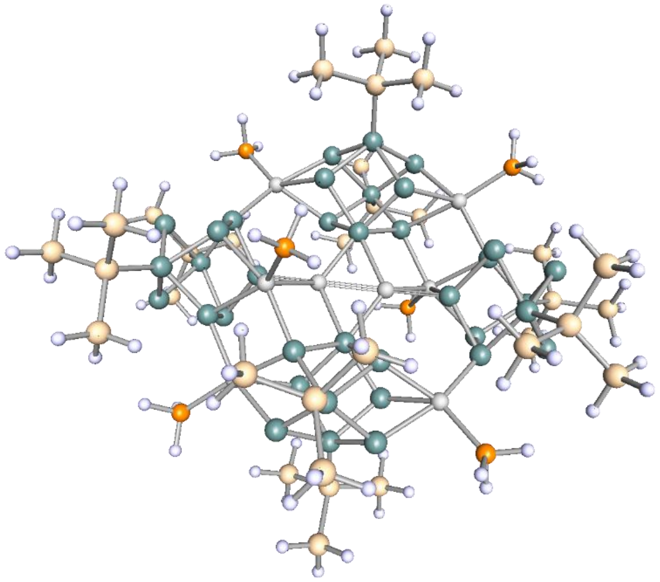

Figure S7: Geometry optimized structure

Point group:	C_1
Energy:	-87314. 90850003576 Hartree
HOMO-LUMO-gap:	1.727 eV

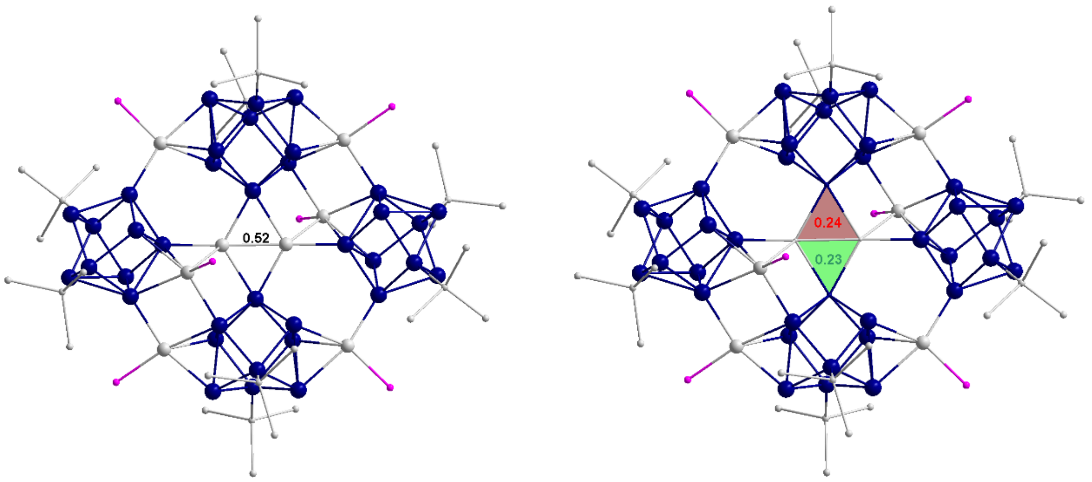

Figure S8: Visual presentation of the 2c-SEN of the central Ag-Ag contact (left) and the 3c-SEN including the top of the coordinated Ge_9Hyp_2 cluster (right)

Table S6: Atomic coordinates:

Ag 9.286926 8.583169 10.232840
Ge 8.177749 8.274224 12.748584
Ge 10.567697 6.703517 11.802695
Ge 7.987944 6.153296 10.739483
Ge 12.237892 7.739438 7.692723
Ge 9.673936 8.114529 7.571463
P 9.965667 11.067539 10.089456
Ag 8.140325 5.673658 7.973063
Ag 10.214490 3.773969 8.867809
Ge 8.795769 5.952789 5.219607
Ge 10.743884 5.630135 7.229872
P 5.765937 4.787549 7.831052
Ag 12.096179 2.772702 4.280419
Ge 12.532684 -0.017419 4.464185
Ge 10.153538 1.176891 5.490995
Ge 12.643970 1.752121 6.781962
Ge 11.183697 5.203618 4.709698
P 13.332178 2.914703 2.018656
Ag 9.867069 1.763041 10.658290
Ge 9.673180 4.299097 11.460724
Ge 10.424997 1.238471 8.061457
Ge 9.756464 6.938830 14.243152
Ge 7.757392 5.580275 15.144923
Ge 10.074276 4.354983 14.016633
Si 11.360887 7.964947 15.729434
Ge 6.622963 6.246401 12.939469
Ge 7.520772 3.806129 12.850708
Si 4.231128 6.530501 12.796043
Ge 12.659458 7.283739 5.167793
Ge 11.412089 9.483942 5.698840
Ge 10.748034 7.562735 3.418698
Si 14.836920 7.579249 4.156495
Ge 9.072574 8.495133 5.120262
Si 7.310272 9.997660 4.460857
Si 3.435573 7.273582 14.890355
Si 3.172311 4.494736 12.249952
Si 3.705681 8.155547 11.169478
Si 10.257878 9.499226 17.142589
Si 12.997583 9.096282 14.462055
Si 12.412499 6.301958 17.031557
Si 6.525490 11.163224 6.354478
Si 5.548332 8.726655 3.540217
Si 8.125940 11.528510 2.861151
Si 14.660354 7.931957 1.831178
Si 16.114416 5.637003 4.559579
Si 15.907193 9.433589 5.148849
Ge 10.516597 -1.403730 5.287800
Ge 13.583032 -0.675316 6.709357
Ge 12.044057 -2.721287 6.850862
Ge 9.595614 -1.185256 7.691076
Si 8.998895 -2.456133 3.730142
Ge 12.120095 -0.590719 8.842358
Si 15.960700 -0.909825 7.044123
Ag 10.838308 -3.045856 9.315298
Si 10.198991 -3.933389 2.335434
Si 7.357124 -3.658288 4.923486
Si 7.940328 -0.807418 2.415522
Si 16.911856 -1.929860 5.140287
Si 16.962534 1.197839 7.384374
Si 16.358088 -2.268432 8.930649
Ge 7.926665 -2.197135 11.915632
Ge 10.500847 -2.545948 11.969755
P 10.087420 -5.508846 9.446775
Ag 12.027182 -0.035631 11.526600
Ge 9.429070 -0.081523 12.337460
Ge 7.575679 -1.733696 14.449337
Ge 8.803421 -3.936396 13.877933
Ge 11.154413 -2.947083 14.403251
Ge 11.436025 -0.407137 14.278903
P 14.267673 1.134793 11.862136
Ge 9.066631 0.346666 14.869648
Ge 9.526743 -2.013599 16.148757
Si 5.427355 -2.005845 15.526149
Si 12.947476 -4.441622 14.992271
Ag 8.174769 2.780263 15.321997
Si 5.669493 -2.358842 17.845565
Si 4.155624 -0.052252 15.161741
Si 4.310321 -3.850340 14.567655
Si 13.483838 -5.792408 13.135421
Si 14.829070 -3.154625 15.600363
Si 12.284448 -5.799561 16.804764
P 7.028227 2.659908 17.630956
H 10.877279 -5.001086 3.159082
H 9.281653 -4.613685 1.345794
H 11.256756 -3.186063 1.560579
H 5.793985 3.350004 17.882401
H 6.692410 1.440853 18.315264
H 7.767493 3.255868 18.708481
H 9.356683 11.911874 9.100679
H 11.326974 11.326925 9.717657
H 9.900245 12.004794 11.179406
H 16.253751 5.382773 6.038861
H 15.444009 4.435620 3.933694
H 17.496828 5.755376 3.961236
H 13.396929 -6.745707 17.191440
H 11.072598 -6.616616 16.432031
H 11.946213 -4.947000 18.002044
H 14.511713 -2.280274 16.787754
H 15.253048 -2.268736 14.454022
H 15.995374 -4.044171 15.961594
H 5.416941 12.118110 5.978501
H 5.994562 10.205728 7.391943
H 7.639804 11.969245 6.978310
H 2.789283 -0.161328 15.797742
H 3.977561 0.207424 13.687585
H 4.852042 1.142384 15.772077
H 12.634098 2.322037 0.912215
H 13.690269 4.137225 1.351909
H 14.576100 2.227885 1.809346
H 15.317651 0.480332 12.589952
H 14.256741 2.346519 12.630705
H 15.075177 1.616570 10.776470
H 15.825109 -1.609799 10.180469
H 15.684241 -3.609204 8.778616
H 17.838667 -2.500101 9.123200
H 15.158057 10.704730 4.834965
H 15.970287 9.266745 6.646787
H 17.318625 9.572492 4.627406
H 6.451997 -4.414779 3.978693
H 8.003480 -4.662619 5.847409
H 6.506610 -2.727411 5.751142
H 6.401289 -1.200220 18.481543
H 6.444298 -3.622530 18.120538
H 4.320202 -2.469590 18.519191
H 16.680910 -1.075037 3.918007
H 16.291814 -3.284340 4.901661
H 18.401989 -2.112673 5.311969
H 16.028062 8.056945 1.198669
H 13.959300 6.766965 1.172668
H 13.881375 9.188131 1.534314
H 18.456171 1.060463 7.569690
H 16.401746 1.853006 8.624163
H 16.714211 2.110332 6.209904
H 5.395770 3.737150 8.734942
H 4.661626 5.658376 8.115839
H 5.227951 4.198393 6.635778
H 4.413158 9.622111 3.102687
H 5.011788 7.756123 4.564457
H 6.026891 7.943672 2.343290
H 3.672780 3.974398 10.923644
H 1.676051 4.677718 12.140867
H 3.445067 3.451006 13.303408
H 10.149902 -6.452299 8.362045
H 8.710013 -5.724273 9.785503
H 10.644826 -6.367894 10.453114
H 9.248283 12.348560 3.446926
H 8.641764 10.794874 1.648608
H 7.028556 12.468510 2.420159
H 13.787661 8.126066 13.619891
H 12.352253 10.108571 13.546377
H 13.959532 9.834862 15.363950
H 13.937263 -4.948708 11.970308
H 12.279412 -6.593184 12.700680
H 14.589278 -6.763827 13.476650
H 4.129175 7.695353 9.795056
H 4.404349 9.458929 11.466910
H 2.217230 8.411543 11.132698
H 13.221143 5.373678 16.159995
H 11.388064 5.487463 17.782891
H 13.342715 6.931182 18.043198
H 8.964112 0.042986 1.704063
H 7.080704 0.087862 3.272446
H 7.059141 -1.450798 1.369429
H 4.092397 8.576278 15.274254
H 3.738814 6.250960 15.958299
H 1.939917 7.487237 14.856346
H 9.585861 10.576660 16.326521
H 9.204383 8.797770 17.964625
H 11.230627 10.163295 18.089369
H 4.199758 -3.681560 13.072664
H 5.058776 -5.127876 14.856103
H 2.915471 -3.978265 15.134644

Paboon results:

The given atom numbers correpond to the position in the atom coordinates list

Table S7: 2c-SEN

shared electron number for the pair	1 ag - 2 ge =	0.4187
shared electron number for the pair	1 ag - 3 ge =	0.3844
shared electron number for the pair	1 ag - 4 ge =	0.3853
shared electron number for the pair	1 ag - 5 ge =	0.0150
shared electron number for the pair	1 ag - 6 ge =	0.1873
shared electron number for the pair	1 ag - 7 p =	0.1820
shared electron number for the pair	1 ag - 20 ge =	-0.0139
shared electron number for the pair	1 ag - 22 ge =	-0.0125
shared electron number for the pair	1 ag - 26 ge =	-0.0121
shared electron number for the pair	2 ge - 3 ge =	0.6066
shared electron number for the pair	2 ge - 4 ge =	0.7056
shared electron number for the pair	2 ge - 7 p =	0.0145
shared electron number for the pair	2 ge - 20 ge =	-0.0153
shared electron number for the pair	2 ge - 22 ge =	1.0400
shared electron number for the pair	2 ge - 23 ge =	0.0868
shared electron number for the pair	2 ge - 24 ge =	-0.0205
shared electron number for the pair	2 ge - 26 ge =	1.0280
shared electron number for the pair	2 ge - 27 ge =	-0.0228
shared electron number for the pair	3 ge - 4 ge =	0.8272
shared electron number for the pair	3 ge - 5 ge =	0.0166
shared electron number for the pair	3 ge - 6 ge =	0.0206
shared electron number for the pair	3 ge - 8 ag =	0.0177
shared electron number for the pair	3 ge - 20 ge =	1.0189
shared electron number for the pair	3 ge - 22 ge =	1.0023
shared electron number for the pair	3 ge - 23 ge =	-0.0219
shared electron number for the pair	3 ge - 24 ge =	0.2615
shared electron number for the pair	3 ge - 27 ge =	-0.0221
shared electron number for the pair	4 ge - 6 ge =	0.0655
shared electron number for the pair	4 ge - 9 ag =	0.0900
shared electron number for the pair	4 ge - 11 ge =	0.0213
shared electron number for the pair	4 ge - 12 p =	0.0276
shared electron number for the pair	4 ge - 19 ag =	0.0293
shared electron number for the pair	4 ge - 20 ge =	1.0351
shared electron number for the pair	4 ge - 22 ge =	0.0186
shared electron number for the pair	4 ge - 23 ge =	-0.0211
shared electron number for the pair	4 ge - 24 ge =	-0.0194
shared electron number for the pair	4 ge - 26 ge =	0.9457
shared electron number for the pair	4 ge - 27 ge =	0.3196
shared electron number for the pair	5 ge - 6 ge =	1.1050
shared electron number for the pair	5 ge - 10 ge =	-0.0175
shared electron number for the pair	5 ge - 11 ge =	0.9295
shared electron number for the pair	5 ge - 29 ge =	0.9280
shared electron number for the pair	5 ge - 30 ge =	0.8049
shared electron number for the pair	5 ge - 31 ge =	-0.0214
shared electron number for the pair	6 ge - 7 p =	0.0274
shared electron number for the pair	6 ge - 8 ag =	0.1548
shared electron number for the pair	6 ge - 9 ag =	0.0366
shared electron number for the pair	6 ge - 10 ge =	0.3607
shared electron number for the pair	6 ge - 11 ge =	0.9290
shared electron number for the pair	6 ge - 17 ge =	-0.0150
shared electron number for the pair	6 ge - 30 ge =	0.6422
shared electron number for the pair	6 ge - 31 ge =	-0.0170
shared electron number for the pair	6 ge - 33 ge =	1.0308
shared electron number for the pair	8 ag - 9 ag =	0.2457
shared electron number for the pair	8 ag - 10 ge =	0.1353
shared electron number for the pair	8 ag - 11 ge =	0.4064
shared electron number for the pair	8 ag - 12 p =	0.0484
shared electron number for the pair	8 ag - 19 ag =	0.0230
shared electron number for the pair	8 ag - 20 ge =	0.0787
shared electron number for the pair	8 ag - 21 ge =	0.0235
shared electron number for the pair	9 ag - 10 ge =	0.0585
shared electron number for the pair	9 ag - 11 ge =	0.7681
shared electron number for the pair	9 ag - 12 p =	0.0143
shared electron number for the pair	9 ag - 16 ge =	0.0208
shared electron number for the pair	9 ag - 19 ag =	0.5260
shared electron number for the pair	9 ag - 20 ge =	0.4276
shared electron number for the pair	9 ag - 21 ge =	0.4060
shared electron number for the pair	9 ag - 27 ge =	0.0125
shared electron number for the pair	9 ag - 52 ge =	0.0322
shared electron number for the pair	9 ag - 64 ag =	0.0270
shared electron number for the pair	9 ag - 65 ge =	0.0541
shared electron number for the pair	10 ge - 11 ge =	0.8102
shared electron number for the pair	10 ge - 12 p =	0.0187
shared electron number for the pair	10 ge - 17 ge =	1.1298
shared electron number for the pair	10 ge - 30 ge =	-0.0210
shared electron number for the pair	10 ge - 31 ge =	0.4085
shared electron number for the pair	10 ge - 33 ge =	1.0354
shared electron number for the pair	11 ge - 16 ge =	0.0240
shared electron number for the pair	11 ge - 17 ge =	0.9919
shared electron number for the pair	11 ge - 19 ag =	0.0540
shared electron number for the pair	11 ge - 20 ge =	0.0147
shared electron number for the pair	11 ge - 21 ge =	0.0199
shared electron number for the pair	11 ge - 29 ge =	0.1916
shared electron number for the pair	11 ge - 30 ge =	-0.0199
shared electron number for the pair	11 ge - 31 ge =	-0.0219
shared electron number for the pair	11 ge - 33 ge =	0.0232
shared electron number for the pair	13 ag - 14 ge =	0.2936
shared electron number for the pair	13 ag - 15 ge =	0.3545
shared electron number for the pair	13 ag - 16 ge =	0.3487
shared electron number for the pair	13 ag - 17 ge =	0.3116
shared electron number for the pair	13 ag - 18 p =	0.1677
shared electron number for the pair	13 ag - 21 ge =	-0.0114
shared electron number for the pair	13 ag - 47 ge =	-0.0108
shared electron number for the pair	14 ge - 15 ge =	0.7946
shared electron number for the pair	14 ge - 16 ge =	0.7163
shared electron number for the pair	14 ge - 18 p =	0.0314
shared electron number for the pair	14 ge - 21 ge =	-0.014
shared electron number for the pair	14 ge - 47 ge =	0.9851
shared electron number for the pair	14 ge - 48 ge =	1.0148
shared electron number for the pair	14 ge - 49 ge =	0.0831
shared electron number for the pair	14 ge - 50 ge =	-0.0217
shared electron number for the pair	14 ge - 52 ge =	-0.0211
shared electron number for the pair	15 ge - 16 ge =	0.8127
shared electron number for the pair	15 ge - 17 ge =	0.0442
shared electron number for the pair	15 ge - 21 ge =	1.0127
shared electron number for the pair	15 ge - 47 ge =	0.9364
shared electron number for the pair	15 ge - 48 ge =	0.0101
shared electron number for the pair	15 ge - 49 ge =	-0.0203
shared electron number for the pair	15 ge - 50 ge =	0.2530
shared electron number for the pair	15 ge - 52 ge =	-0.0188
shared electron number for the pair	16 ge - 17 ge =	0.0372
shared electron number for the pair	16 ge - 19 ag =	0.0144
shared electron number for the pair	16 ge - 21 ge =	1.0313
shared electron number for the pair	16 ge - 47 ge =	0.0110
shared electron number for the pair	16 ge - 48 ge =	0.9483
shared electron number for the pair	16 ge - 49 ge =	-0.0231
shared electron number for the pair	16 ge - 50 ge =	-0.0220
shared electron number for the pair	16 ge - 52 ge =	0.3409
shared electron number for the pair	17 ge - 18 p =	0.0154
shared electron number for the pair	17 ge - 29 ge =	0.9668
shared electron number for the pair	17 ge - 30 ge =	-0.0224
shared electron number for the pair	17 ge - 31 ge =	0.8669
shared electron number for the pair	17 ge - 33 ge =	0.0102
shared electron number for the pair	19 ag - 20 ge =	0.4014
shared electron number for the pair	19 ag - 21 ge =	0.4195
shared electron number for the pair	19 ag - 27 ge =	0.0197
shared electron number for the pair	19 ag - 52 ge =	0.0924
shared electron number for the pair	19 ag - 62 ge =	0.0366
shared electron number for the pair	19 ag - 64 ag =	0.2374
shared electron number for the pair	19 ag - 65 ge =	0.7675
shared electron number for the pair	19 ag - 69 ge =	0.0602
shared electron number for the pair	19 ag - 70 p =	0.0157
shared electron number for the pair	20 ge - 21 ge =	0.0105
shared electron number for the pair	20 ge - 22 ge =	0.0161
shared electron number for the pair	20 ge - 23 ge =	-0.0138
shared electron number for the pair	20 ge - 24 ge =	1.0142
shared electron number for the pair	20 ge - 27 ge =	1.0308
shared electron number for the pair	20 ge - 64 ag =	0.0254
shared electron number for the pair	20 ge - 65 ge =	0.0200
shared electron number for the pair	20 ge - 75 ag =	-0.0114
shared electron number for the pair	21 ge - 47 ge =	0.0171
shared electron number for the pair	21 ge - 49 ge =	-0.0160
shared electron number for the pair	21 ge - 50 ge =	1.0188
shared electron number for the pair	21 ge - 52 ge =	1.0289
shared electron number for the pair	21 ge - 54 ag =	-0.0132
shared electron number for the pair	21 ge - 64 ag =	0.0788
shared electron number for the pair	21 ge - 65 ge =	0.0142
shared electron number for the pair	22 ge - 23 ge =	0.9878

shared electron number for the pair 22 ge - 24 ge = 0.9402
shared electron number for the pair 22 ge - 25 si = 1.1246
shared electron number for the pair 22 ge - 26 ge = 0.079
shared electron number for the pair 22 ge - 27 ge = 0.0101
shared electron number for the pair 22 ge - 39 si = 0.0136
shared electron number for the pair 22 ge - 40 si = 0.0133
shared electron number for the pair 22 ge - 75 ag = -0.0106
shared electron number for the pair 23 ge - 24 ge = 0.7955
shared electron number for the pair 23 ge - 26 ge = 1.0142
shared electron number for the pair 23 ge - 27 ge = 0.7224
shared electron number for the pair 23 ge - 75 ag = 0.2882
shared electron number for the pair 23 ge - 82 p = 0.0311
shared electron number for the pair 24 ge - 26 ge = 0.0102
shared electron number for the pair 24 ge - 27 ge = 0.8168
shared electron number for the pair 24 ge - 71 ge = 0.0447
shared electron number for the pair 24 ge - 75 ag = 0.3528
shared electron number for the pair 26 ge - 27 ge = 0.9482
shared electron number for the pair 26 ge - 28 si = 1.1221
shared electron number for the pair 26 ge - 35 si = 0.0133
shared electron number for the pair 26 ge - 37 si = 0.0115
shared electron number for the pair 27 ge - 65 ge = 0.0228
shared electron number for the pair 27 ge - 71 ge = 0.0368
shared electron number for the pair 27 ge - 75 ag = 0.3497
shared electron number for the pair 29 ge - 30 ge = 1.0105
shared electron number for the pair 29 ge - 31 ge = 0.9945
shared electron number for the pair 29 ge - 32 si = 1.1179
shared electron number for the pair 29 ge - 33 ge = 0.0258
shared electron number for the pair 29 ge - 44 si = 0.0119
shared electron number for the pair 29 ge - 45 si = 0.0116
shared electron number for the pair 29 ge - 46 si = 0.0143
shared electron number for the pair 30 ge - 31 ge = 0.4386
shared electron number for the pair 30 ge - 33 ge = 0.9439
shared electron number for the pair 31 ge - 32 si = 0.0158
shared electron number for the pair 31 ge - 33 ge = 1.0117
shared electron number for the pair 31 ge - 44 si = 0.0124
shared electron number for the pair 33 ge - 34 si = 1.1214
shared electron number for the pair 33 ge - 41 si = 0.0121
shared electron number for the pair 33 ge - 42 si = 0.0136
shared electron number for the pair 33 ge - 43 si = 0.0119
shared electron number for the pair 47 ge - 48 ge = 0.0808
shared electron number for the pair 47 ge - 49 ge = 1.0408
shared electron number for the pair 47 ge - 50 ge = 1.0024
shared electron number for the pair 47 ge - 51 si = 1.1236
shared electron number for the pair 47 ge - 52 ge = 0.0197
shared electron number for the pair 47 ge - 54 ag = -0.0126
shared electron number for the pair 47 ge - 56 si = 0.0137
shared electron number for the pair 47 ge - 57 si = 0.0133
shared electron number for the pair 48 ge - 49 ge = 1.0263
shared electron number for the pair 48 ge - 52 ge = 0.9543
shared electron number for the pair 48 ge - 53 si = 1.1222
shared electron number for the pair 48 ge - 54 ag = -0.0122
shared electron number for the pair 48 ge - 58 si = 0.0117
shared electron number for the pair 48 ge - 59 si = 0.0107
shared electron number for the pair 48 ge - 60 si = 0.0129
shared electron number for the pair 49 ge - 50 ge = 0.6085
shared electron number for the pair 49 ge - 52 ge = 0.7114
shared electron number for the pair 49 ge - 54 ag = 0.4230
shared electron number for the pair 49 ge - 63 p = 0.0134
shared electron number for the pair 50 ge - 52 ge = 0.8309
shared electron number for the pair 50 ge - 54 ag = 0.3824
shared electron number for the pair 50 ge - 61 ge = 0.0132
shared electron number for the pair 50 ge - 62 ge = 0.0192
shared electron number for the pair 50 ge - 64 ag = 0.0145
shared electron number for the pair 51 si - 55 si = 1.2936
shared electron number for the pair 52 ge - 54 ag = 0.3717
shared electron number for the pair 52 ge - 62 ge = 0.0716
shared electron number for the pair 52 ge - 64 ag = 0.1696
shared electron number for the pair 52 ge - 65 ge = 0.0233
shared electron number for the pair 52 ge - 70 p = 0.0195
shared electron number for the pair 54 ag - 61 ge = 0.0140
shared electron number for the pair 54 ag - 62 ge = 0.1900
shared electron number for the pair 54 ag - 63 p = 0.1869
shared electron number for the pair 54 ag - 64 ag = 0.0215
shared electron number for the pair 61 ge - 62 ge = 1.0972
shared electron number for the pair 61 ge - 65 ge = 0.9278
shared electron number for the pair 61 ge - 66 ge = 0.9270
shared electron number for the pair 61 ge - 67 ge = 0.8194
shared electron number for the pair 61 ge - 69 ge = -0.0182
shared electron number for the pair 61 ge - 72 ge = -0.0215
shared electron number for the pair 62 ge - 63 p = 0.0259
shared electron number for the pair 62 ge - 64 ag = 0.1473
shared electron number for the pair 62 ge - 65 ge = 0.9508
shared electron number for the pair 62 ge - 66 ge = 0.0100
shared electron number for the pair 62 ge - 67 ge = 0.6336
shared electron number for the pair 62 ge - 68 ge = 1.0325
shared electron number for the pair 62 ge - 69 ge = 0.3841
shared electron number for the pair 62 ge - 71 ge = -0.0155
shared electron number for the pair 62 ge - 72 ge = -0.0170
shared electron number for the pair 64 ag - 65 ge = 0.4144
shared electron number for the pair 64 ag - 69 ge = 0.1410
shared electron number for the pair 64 ag - 70 p = 0.0469
shared electron number for the pair 65 ge - 66 ge = 0.1966
shared electron number for the pair 65 ge - 67 ge = -0.0207
shared electron number for the pair 65 ge - 68 ge = 0.0219
shared electron number for the pair 65 ge - 69 ge = 0.8222
shared electron number for the pair 65 ge - 71 ge = 0.9896
shared electron number for the pair 65 ge - 72 ge = -0.0220
shared electron number for the pair 66 ge - 67 ge = 1.0055
shared electron number for the pair 66 ge - 68 ge = 0.0268
shared electron number for the pair 66 ge - 71 ge = 0.9637
shared electron number for the pair 66 ge - 72 ge = 0.9994
shared electron number for the pair 66 ge - 73 si = 1.1181
shared electron number for the pair 66 ge - 76 si = 0.0119
shared electron number for the pair 66 ge - 77 si = 0.0116
shared electron number for the pair 66 ge - 78 si = 0.0143
shared electron number for the pair 67 ge - 68 ge = 0.9419
shared electron number for the pair 67 ge - 69 ge = -0.0214
shared electron number for the pair 67 ge - 71 ge = -0.0222
shared electron number for the pair 67 ge - 72 ge = 0.4304
shared electron number for the pair 68 ge - 69 ge = 1.0420
shared electron number for the pair 68 ge - 71 ge = 0.0105
shared electron number for the pair 68 ge - 72 ge = 1.0114
shared electron number for the pair 68 ge - 74 si = 1.1218
shared electron number for the pair 68 ge - 79 si = 0.0121
shared electron number for the pair 68 ge - 80 si = 0.0135
shared electron number for the pair 68 ge - 81 si = 0.0121
shared electron number for the pair 69 ge - 70 p = 0.0236
shared electron number for the pair 69 ge - 71 ge = 1.1308
shared electron number for the pair 69 ge - 72 ge = 0.4005

Table S8: 3c-SEN and 4c-SEN

n(1 2 3) = 0.1455
n(1 2 3 4) = 0.0902
n(1 2 3 6) = -0.0170
n(1 2 3 20) = -0.0143
n(1 2 4) = 0.1665
n(1 2 4 6) = -0.0214
n(1 2 4 7) = -0.0239
n(1 2 4 20) = -0.0127
n(1 2 6) = -0.1341
n(1 2 7) = -0.0400
n(1 2 22) = -0.0260
n(1 2 22 23) = -0.0108
n(1 2 23) = -0.0190
n(1 2 23 26) = -0.0108
n(1 2 26) = -0.0307
n(1 3 4) = 0.1643
n(1 3 4 7) = -0.0275
n(1 3 4 8) = 0.0143
n(1 3 6) = -0.0364
n(1 3 7) = -0.0785
n(1 3 8) = 0.0173
n(1 3 8 11) = -0.0115
n(1 3 20) = -0.0357
n(1 3 20 24) = -0.0155
n(1 3 22) = -0.0269
n(1 3 22 24) = -0.0142
n(1 3 24) = -0.0390
n(1 4 6 8) = 0.0139
n(1 4 7) = -0.1382
n(1 4 7 27) = 0.0115
n(1 4 8) = 0.0192
n(1 4 8 9) = -0.0311
n(1 4 20) = -0.0318
n(1 4 20 27) = -0.0136
n(1 4 26) = -0.0295
n(1 4 26 27) = -0.0148
n(1 4 27) = -0.0455
n(1 6 8) = 0.0170
n(1 6 8 9) = 0.0300
n(1 6 8 10) = -0.0178
n(1 6 8 11) = -0.0143
n(1 6 8 33) = -0.0144
n(1 6 10) = -0.0135
n(1 6 30) = -0.0204
n(1 6 30 33) = -0.0116
n(1 6 33) = -0.0462
n(1 7 89) = -0.0118
n(1 7 90) = -0.0101
n(1 7 91) = -0.0134
n(1 8 9) = -0.0338
n(1 8 9 10) = 0.0357
n(1 8 9 11) = 0.0280
n(1 8 9 33) = 0.0158
n(1 8 10) = -0.0194
n(1 8 11) = -0.0391
n(1 8 11 19) = 0.0182
n(1 8 11 20) = 0.0138
n(1 9 11 19) = 0.0113
n(2 3 4) = 0.2249
n(2 3 4 22) = 0.0416
n(2 3 4 26) = 0.0244
n(2 3 20) = -0.0390
n(2 3 20 26) = 0.0217
n(2 3 22) = 0.2169
n(2 3 22 24) = -0.0126
n(2 3 22 26) = 0.0155
n(2 3 23 24) = 0.0114
n(2 3 24) = -0.0136
n(2 3 26 28) = -0.0180
n(2 4 8) = -0.0286
n(2 4 8 9) = -0.0304
n(2 4 8 26) = -0.0148
n(2 4 9) = -0.0235
n(2 4 9 20) = -0.0122
n(2 4 9 26) = -0.0115
n(2 4 12) = -0.0110
n(2 4 19) = -0.0114
n(2 4 20) = -0.0438
n(2 4 20 22) = 0.0237
n(2 4 22) = 0.0177
n(2 4 22 25) = -0.0221
n(2 4 22 26) = 0.0207
n(2 4 23 27) = 0.0129
n(2 4 26) = 0.2305
n(2 4 26 27) = -0.0157
n(2 4 26 28) = -0.0111
n(2 4 27) = -0.0196
n(2 6 8 9) = 0.0106
n(2 22 23) = 0.0510
n(2 22 23 24) = -0.0125
n(2 22 23 26) = 0.0639
n(2 22 24) = -0.0925
n(2 22 25) = -0.0605
n(2 22 25 26) = -0.0324
n(2 22 25 40) = -0.0288
n(2 22 26) = 0.0736
n(2 22 26 28) = -0.0296
n(2 22 40) = -0.0226
n(2 23 26) = 0.0480
n(2 23 26 27) = -0.0111
n(2 23 75) = -0.0165
n(2 26 27) = -0.0932
n(2 26 28) = -0.0496
n(2 26 28 36) = -0.0261
n(2 26 36) = -0.0181
n(2 28 36) = -0.0107
n(3 4 6) = 0.0120
n(3 4 8) = 0.0182
n(3 4 8 20) = 0.0215
n(3 4 9) = 0.0160
n(3 4 9 20) = 0.0140
n(3 4 12) = -0.0120
n(3 4 20) = 0.2761
n(3 4 20 22) = 0.0129
n(3 4 22) = 0.0136
n(3 4 22 25) = -0.0256
n(3 4 22 26) = 0.0428
n(3 4 24 27) = 0.0246
n(3 4 26 28) = -0.0232
n(3 4 27) = -0.0113
n(3 6 8 9) = 0.0224
n(3 6 33) = -0.0136
n(3 8 9 20) = 0.0202
n(3 8 9 22) = -0.0117
n(3 8 11) = -0.0212
n(3 8 20) = 0.0200
n(3 8 22) = -0.0160
n(3 9 19 20) = -0.0191
n(3 9 20) = -0.0163
n(3 9 20 22) = -0.0149
n(3 9 20 27) = -0.0102
n(3 9 22) = -0.0120
n(3 19 20) = -0.0305
n(3 19 20 22) = -0.0117
n(3 19 20 65) = -0.0143
n(3 20 22) = 0.0120
n(3 20 22 24) = 0.0611
n(3 20 22 25) = -0.0210
n(3 20 22 26) = 0.0104
n(3 20 22 27) = 0.0135
n(3 20 24) = 0.1446
n(3 20 24 75) = -0.0126
n(3 20 26) = 0.0139
n(3 20 26 27) = 0.0118
n(3 20 26 28) = -0.0127
n(3 20 27) = -0.0699
n(3 20 65) = -0.0107
n(3 22 23) = -0.0923
n(3 22 23 24) = -0.0150
n(3 22 24) = 0.1394
n(3 22 24 75) = -0.0170
n(3 22 25) = -0.0311
n(3 22 25 38) = -0.0204
n(3 22 25 39) = 0.0106
n(3 22 26) = 0.0158
n(3 22 26 28) = -0.0169
n(3 22 38) = -0.0136
n(3 23 24) = -0.0189
n(3 24 75) = -0.0386
n(3 26 28) = -0.0297
n(4 5 8 9) = 0.0134
n(4 6 8) = -0.0102
n(4 6 8 9) = 0.1418
n(4 6 8 11) = 0.0267
n(4 6 8 20) = -0.0174
n(4 6 8 26) = -0.0122
n(4 6 8 30) = -0.0112
n(4 6 8 33) = -0.0149
n(4 6 9) = 0.0153
n(4 6 9 11) = 0.0139
n(4 6 11) = 0.0123
n(4 6 26) = -0.0225
n(4 6 27) = -0.0116
n(4 6 30) = -0.0199
n(4 6 33) = -0.0158
n(4 8 9 10) = 0.0643
n(4 8 9 11) = -0.0515
n(4 8 9 19) = 0.0770
n(4 8 9 20) = 0.0662
n(4 8 9 21) = -0.0217
n(4 8 9 26) = -0.0398
n(4 8 9 33) = 0.0163
n(4 8 10) = -0.0728
n(4 8 10 19) = 0.0149
n(4 8 10 20) = -0.0135
n(4 8 11) = -0.1336
n(4 8 11 17) = -0.0105
n(4 8 11 19) = 0.0347
n(4 8 11 20) = -0.0139
n(4 8 11 21) = 0.0175
n(4 8 19) = 0.0141
n(4 8 19 20) = 0.0264
n(4 8 19 21) = -0.0202
n(4 8 19 26) = -0.0114
n(4 8 19 65) = -0.0116
n(4 8 20) = 0.0492
n(4 8 20 24) = -0.0179
n(4 8 20 26) = -0.0108
n(4 8 21) = -0.0136
n(4 8 26) = -0.0660
n(4 9 11) = -0.0658
n(4 9 11 19) = -0.0123
n(4 9 11 20) = -0.0225
n(4 9 19) = -0.0488
n(4 9 19 20) = -0.0390
n(4 9 19 64) = -0.0128
n(4 9 19 65) = -0.0180
n(4 9 20) = 0.0415
n(4 9 20 21) = -0.0135
n(4 9 20 24) = -0.0169
n(4 9 20 26) = -0.0153
n(4 9 20 64) = -0.0140
n(4 9 21) = -0.0372
n(4 9 26) = -0.0386
n(4 11 26) = -0.0152
n(4 12136) = -0.0157
n(4 19 20 24) = -0.0104
n(4 19 20 26) = -0.0114
n(4 19 20 27) = 0.0115
n(4 19 20 64) = -0.0201
n(4 19 20 65) = -0.0365
n(4 19 21) = -0.0220
n(4 19 64) = -0.0179
n(4 19 64 65) = 0.0125
n(4 19 65) = -0.0361
n(4 20 22) = 0.0197
n(4 20 22 24) = 0.0174
n(4 20 22 25) = -0.0155
n(4 20 22 26) = 0.0106
n(4 20 24) = -0.0628
n(4 20 24 26) = 0.0166
n(4 20 26 27) = 0.0593
n(4 20 26 28) = -0.0239
n(4 20 27) = 0.1822
n(4 20 27 75) = -0.0122
n(4 20 64) = -0.0107
n(4 22 24 27) = 0.0102
n(4 22 25) = -0.0394
n(4 22 25 26) = -0.0205
n(4 22 26) = 0.0202
n(4 23 24 26) = 0.0104
n(4 23 26) = -0.0861
n(4 23 26 27) = -0.0109
n(4 23 27) = -0.0155
n(4 26 27) = 0.1563
n(4 26 27 75) = -0.0176
n(4 26 28) = -0.0491
n(4 26 28 35) = -0.0267
n(4 26 35) = -0.0197
n(4 27 75) = -0.0429
n(4 28 35) = -0.0103
n(5 6 8) = 0.0117
n(5 6 8 11) = 0.0325
n(5 6 9) = 0.0106
n(5 6 9 19) = -0.0263
n(5 6 10) = -0.0401
n(5 6 10 11) = -0.0134
n(5 6 10 17) = 0.0125
n(5 6 11) = 0.2747
n(5 6 11 29) = 0.0242
n(5 6 29 30) = 0.0330
n(5 6 29 32) = -0.0317
n(5 6 29 33) = 0.0300
n(5 6 30) = 0.2677
n(5 6 30 33) = 0.0124
n(5 6 33) = -0.0513
n(5 6 33 34) = -0.0244
n(5 8 9) = -0.0263
n(5 8 9 11) = -0.0442
n(5 8 10) = -0.0130
n(5 8 10 11) = -0.0240
n(5 8 11) = -0.0187
n(5 9 10) = -0.0107
n(5 9 10 11) = -0.0214
n(5 9 10 19) = 0.0125
n(5 9 11) = -0.0449
n(5 9 11 19) = -0.0434
n(5 9 11 29) = -0.0164
n(5 9 11 30) = -0.0120
n(5 9 19) = -0.0627
n(5 9 19 65) = 0.0180
n(5 10 11) = -0.0645
n(5 10 11 17) = -0.0152
n(5 11 17) = -0.0180
n(5 11 17 29) = 0.0423
n(5 11 29) = 0.1101
n(5 11 29 32) = -0.0228
n(5 11 30) = -0.0431
n(5 11 30 33) = 0.0183
n(5 11 33) = 0.0111
n(5 11 33 34) = -0.0116
n(5 17 29) = -0.0215
n(5 17 29 30) = -0.0124
n(5 17 29 31) = -0.0162
n(5 29 30) = 0.2271
n(5 29 30 31) = -0.0277
n(5 29 31) = -0.0965
n(5 29 32) = -0.0547
n(5 29 32 44) = -0.0241
n(5 29 33) = 0.0132
n(5 29 33 34) = -0.0123
n(5 29 44) = -0.0174
n(5 30 31) = -0.0398
n(5 30 31 33) = -0.0113
n(5 30 33) = -0.0258
n(5 30 33 34) = -0.0169
n(5 33 34) = -0.0250
n(6 7 11) = -0.0113
n(6 7 91) = -0.0156
n(6 8 9) = -0.1953
n(6 8 9 10) = 0.0257
n(6 8 9 11) = -0.1130
n(6 8 9 12) = 0.0198
n(6 8 9 19) = -0.0706
n(6 8 9 20) = -0.0190
n(6 8 9 33) = -0.0300
n(6 8 10) = 0.0405
n(6 8 10 11) = 0.0520
n(6 8 10 33) = 0.0193
n(6 8 11) = 0.0607
n(6 8 11 17) = -0.0261
n(6 8 11 19) = -0.0361
n(6 8 11 21) = -0.0132
n(6 8 12) = -0.0173
n(6 8 17) = -0.0122
n(6 8 19) = -0.0552
n(6 8 19 20) = -0.0180
n(6 8 19 65) = 0.0259
n(6 8 20) = -0.0371
n(6 8 21) = -0.0114
n(6 8 30) = -0.0329
n(6 8 33) = -0.0144
n(6 9 10) = 0.0229
n(6 9 10 11) = 0.0229
n(6 9 10 19) = -0.0155
n(6 9 11 17) = -0.0180
n(6 9 11 21) = -0.0104
n(6 9 11 30) = -0.0104
n(6 9 17) = -0.0111
n(6 9 17 19) = 0.0115
n(6 9 19) = -0.0319
n(6 9 21) = -0.0128
n(6 9 30) = -0.0122
n(6 9 33) = -0.0101
n(6 10 11) = 0.1943
n(6 10 11 33) = 0.0618
n(6 10 17) = -0.0184
n(6 10 17 29) = 0.0151
n(6 10 29) = 0.0103
n(6 10 30) = -0.0140
n(6 10 30 31) = 0.0178
n(6 10 30 33) = -0.0134
n(6 10 33) = 0.1592
n(6 11 13 17) = -0.0110
n(6 11 16) = -0.0127
n(6 11 17) = -0.0485
n(6 11 17 33) = 0.0227
n(6 11 29) = 0.0240
n(6 11 29 30) = 0.0411
n(6 11 29 32) = -0.0240
n(6 11 29 33) = 0.0131
n(6 11 30) = -0.0223
n(6 11 33) = 0.0148
n(6 11 33 34) = -0.0293
n(6 29 30) = 0.0146
n(6 29 30 32) = -0.0207
n(6 29 30 33) = 0.0133
n(6 29 31 33) = 0.0176
n(6 29 32) = -0.0475
n(6 29 32 33) = -0.0181
n(6 29 33) = 0.0221
n(6 30 31) = -0.0164
n(6 30 31 33) = -0.0138
n(6 30 33) = 0.2252
n(6 30 33 34) = -0.0105
n(6 31 33) = -0.0835
n(6 33 34) = -0.0684
n(6 33 34 42) = -0.0125
n(6 33 34 43) = -0.0185
n(6 33 42) = -0.0103
n(6 33 43) = -0.0139
n(8 9 10) = -0.0270
n(8 9 10 11) = -0.0542
n(8 9 10 19) = -0.0457
n(8 9 10 20) = -0.0242
n(8 9 10 33) = -0.0451
n(8 9 11) = 0.0983
n(8 9 11 12) = -0.0284
n(8 9 11 17) = -0.0531
n(8 9 11 19) = 0.0400
n(8 9 11 20) = -0.0599
n(8 9 11 29) = -0.0409
n(8 9 11 33) = -0.0238
n(8 9 11 52) = 0.0129
n(8 9 11 64) = 0.0215
n(8 9 17) = -0.0231
n(8 9 19) = -0.0111
n(8 9 19 20) = 0.0719
n(8 9 19 21) = -0.0229
n(8 9 19 52) = -0.0170
n(8 9 19 64) = -0.0335
n(8 9 19 65) = -0.0714
n(8 9 20) = 0.0637
n(8 9 20 24) = -0.0344
n(8 9 20 27) = -0.0133
n(8 9 21) = -0.0247
n(8 9 21 52) = -0.0229
n(8 9 24) = -0.0105
n(8 9 30) = 0.0189
n(8 9 33) = -0.0191
n(8 9 33 34) = 0.0125
n(8 9 52) = -0.0213
n(8 9 64) = -0.0162
n(8 9 65) = -0.0170
n(8 10 11) = 0.0586
n(8 10 11 17) = 0.0226
n(8 10 11 19) = -0.0307
n(8 10 11 20) = -0.0154
n(8 10 19) = -0.0320
n(8 10 19 20) = -0.0137
n(8 10 19 65) = 0.0134
n(8 10 20) = -0.0312
n(8 10 31) = -0.0172
n(8 11 12) = -0.0447
n(8 11 12 20) = 0.0122
n(8 11 17) = -0.0449
n(8 11 19) = -0.0259
n(8 11 19 20) = -0.0365
n(8 11 19 21) = -0.0190
n(8 11 20) = -0.0680
n(8 11 21) = -0.0166
n(8 11 29) = -0.0365
n(8 12135) = -0.0127
n(8 12136) = -0.0129
n(8 19 20) = 0.0518

n(8 19 20 21) = 0.0253
n(8 19 20 24) = -0.0180
n(8 19 20 65) = -0.0167
n(8 19 21 52) = -0.0138
n(8 19 64) = -0.0127
n(8 19 64 65) = 0.0224
n(8 19 65) = -0.0574
n(8 20 24) = -0.0299
n(9 10 11) = 0.0161
n(9 10 11 17) = 0.0161
n(9 10 11 19) = 0.0206
n(9 10 17) = 0.0141
n(9 10 17 19) = -0.0246
n(9 10 19) = -0.0238
n(9 10 20) = -0.0119
n(9 10 31) = -0.0102
n(9 10 33) = -0.0107
n(9 11 12) = -0.0310
n(9 11 17) = -0.0500
n(9 11 17 19) = -0.0272
n(9 11 17 29) = -0.0174
n(9 11 17 31) = -0.0130
n(9 11 19) = -0.2463
n(9 11 19 20) = -0.0493
n(9 11 19 21) = -0.0275
n(9 11 19 52) = -0.0166
n(9 11 19 61) = 0.0175
n(9 11 19 64) = -0.0723
n(9 11 19 65) = 0.0164
n(9 11 19 71) = 0.0112
n(9 11 20) = -0.0627
n(9 11 20 21) = 0.0171
n(9 11 20 24) = -0.0131
n(9 11 21) = -0.0417
n(9 11 21 50) = -0.0166
n(9 11 21 52) = -0.0387
n(9 11 21 64) = -0.0147
n(9 11 29) = -0.0619
n(9 11 50) = -0.0115
n(9 11 52) = -0.0401
n(9 11 52 64) = -0.0129
n(9 11 62 64) = 0.0267
n(9 11 64) = -0.0617
n(9 11 64 69) = 0.0166
n(9 11 65) = -0.0335
n(9 12 19) = -0.0148
n(9 15 19) = -0.0149
n(9 15 19 21) = -0.0393
n(9 15 21) = -0.0298
n(9 15 21 47) = -0.0118
n(9 15 21 64) = -0.0192
n(9 16 19 20) = -0.0183
n(9 16 19 65) = 0.0117
n(9 16 20) = -0.0214
n(9 16 20 21) = -0.0113
n(9 16 21 52) = 0.0132
n(9 16 52) = 0.0107
n(9 17 19) = -0.0574
n(9 17 19 29) = -0.0133
n(9 17 19 65) = 0.0147
n(9 19 20) = 0.2424
n(9 19 20 21) = -0.2294
n(9 19 20 24) = -0.0445
n(9 19 20 64) = -0.0161
n(9 19 20 65) = -0.0298
n(9 19 21) = 0.2317
n(9 19 21 27) = -0.0157
n(9 19 21 47) = -0.0102
n(9 19 21 50) = -0.0257
n(9 19 21 52) = -0.0479
n(9 19 21 64) = 0.0712
n(9 19 21 65) = -0.0437
n(9 19 24) = -0.0212
n(9 19 29) = -0.0107
n(9 19 50) = -0.0113
n(9 19 52) = -0.0662
n(9 19 52 64) = 0.0652
n(9 19 52 65) = -0.0141
n(9 19 61) = -0.0619
n(9 19 61 62) = -0.0245
n(9 19 61 65) = -0.0413
n(9 19 61 69) = 0.0107
n(9 19 62) = -0.0286
n(9 19 62 64) = -0.0600
n(9 19 62 69) = -0.0139
n(9 19 62 71) = 0.0102
n(9 19 64 65) = 0.0247
n(9 19 64 69) = -0.0469
n(9 19 65) = -0.2448
n(9 19 65 69) = 0.0215
n(9 19 65 70) = -0.0104
n(9 19 65 71) = -0.0229
n(9 19 65 75) = 0.0103
n(9 19 66) = -0.0110
n(9 19 66 71) = -0.0133
n(9 19 69) = -0.0212
n(9 19 69 71) = -0.0209
n(9 19 70) = -0.0221
n(9 19 71) = -0.0518
n(9 20 21) = -0.3424
n(9 20 21 27) = -0.0127
n(9 20 21 64) = 0.0259
n(9 20 21 65) = 0.0212
n(9 20 22) = -0.0119
n(9 20 22 24) = -0.0124
n(9 20 24) = -0.0515
n(9 20 52) = -0.0216
n(9 20 52 64) = -0.0201
n(9 20 64 65) = -0.0198
n(9 21 27) = -0.0230
n(9 21 47) = -0.0105
n(9 21 47 50) = -0.0119
n(9 21 48 52) = -0.0112
n(9 21 49 52) = -0.0101
n(9 21 50) = -0.0316
n(9 21 52) = 0.0102
n(9 21 52 64) = 0.0284
n(9 21 62 64) = -0.0170
n(9 21 64) = 0.0507
n(9 21 64 65) = -0.0387
n(9 21 64 69) = -0.0145
n(9 21 65) = -0.0133
n(9 48 52) = -0.0100
n(9 48 52 64) = -0.0118
n(9 49 52) = -0.0123
n(9 49 52 64) = -0.0104
n(9 52 64) = 0.0159
n(9 52 64 65) = 0.0349
n(9 52 64 69) = 0.0151
n(9 54 64 65) = 0.0163
n(9 62 64) = -0.0552
n(9 62 64 65) = -0.0368
n(9 64 65) = -0.0314
n(9 64 65 69) = -0.0338
n(9 64 69) = -0.0379
n(10 11 17) = 0.2785
n(10 11 17 29) = 0.0186
n(10 11 17 33) = 0.0134
n(10 11 29) = 0.0125
n(10 11 29 31) = 0.0325
n(10 11 29 32) = -0.0178
n(10 11 29 33) = 0.0144
n(10 11 31) = -0.0106
n(10 11 33) = 0.0227
n(10 11 33 34) = -0.0245
n(10 13 17) = -0.0255
n(10 13 17 33) = -0.0109
n(10 17 29) = -0.0289
n(10 17 29 31) = 0.0143
n(10 17 29 32) = -0.0289
n(10 17 29 33) = 0.0303
n(10 17 31) = 0.2131
n(10 17 31 33) = 0.0488
n(10 17 33 34) = -0.0294
n(10 29 30 33) = 0.0113
n(10 29 31 32) = -0.0131
n(10 29 31 33) = 0.0115
n(10 29 32) = -0.0325
n(10 29 32 33) = -0.0146
n(10 29 33) = 0.0194
n(10 30 31) = -0.0127
n(10 30 31 33) = -0.0126
n(10 30 33) = -0.0910
n(10 31 33) = 0.1702
n(10 33 34) = -0.0627
n(10 33 34 43) = -0.0194
n(10 33 43) = -0.0148
n(11 16 17) = 0.0118
n(11 16 48) = -0.0140
n(11 17 29) = 0.1293
n(11 17 29 32) = -0.0270
n(11 17 31) = -0.0517
n(11 17 31 33) = 0.0173
n(11 17 33) = 0.0186
n(11 17 33 34) = -0.0155
n(11 19 20) = -0.0123
n(11 19 20 21) = 0.0224
n(11 19 64) = -0.0159
n(11 19 65) = -0.0324
n(11 21 50) = -0.0108
n(11 29 31) = -0.0161
n(11 29 32) = -0.0715
n(11 33 34) = -0.0434
n(13 14 15) = 0.1434
n(13 14 15 16) = 0.0811
n(13 14 15 17) = -0.0394
n(13 14 15 18) = -0.0136
n(13 14 15 21) = -0.0119
n(13 14 16) = 0.1306
n(13 14 16 17) = -0.0371
n(13 14 16 18) = -0.0119
n(13 14 16 21) = -0.0110
n(13 14 17) = -0.1833
n(13 14 47) = -0.0291
n(13 14 48) = -0.0261
n(13 14 49) = -0.0163
n(13 15 16) = 0.1460
n(13 15 16 18) = -0.0227
n(13 15 16 47) = -0.0111
n(13 15 16 48) = -0.0107
n(13 15 17) = -0.0327
n(13 15 17 18) = 0.0207
n(13 15 18) = -0.0968
n(13 15 21) = -0.0242
n(13 15 21 50) = -0.0126
n(13 15 47) = -0.0406
n(13 15 47 50) = -0.0169
n(13 15 50) = -0.0379
n(13 16 17) = -0.0489
n(13 16 17 18) = 0.0224
n(13 16 18) = -0.0898
n(13 16 21) = -0.0232
n(13 16 21 52) = -0.0121
n(13 16 48) = -0.0372
n(13 16 48 52) = -0.0177
n(13 16 52) = -0.0442
n(13 17 18) = -0.0276
n(13 17 29) = -0.0281
n(13 17 29 31) = -0.0133
n(13 17 31) = -0.0362
n(13 17 33) = -0.0101
n(13 18108) = -0.0141
n(13 18109) = -0.0123
n(14 15 16) = 0.2532
n(14 15 16 47) = 0.0324
n(14 15 16 48) = 0.0317
n(14 15 21) = -0.0423
n(14 15 21 48) = 0.0230
n(14 15 47) = 0.2520
n(14 15 47 48) = 0.0164
n(14 15 47 49) = -0.0125
n(14 15 47 50) = -0.0150
n(14 15 48 52) = 0.0106
n(14 15 48 53) = -0.0222
n(14 15 49 50) = 0.0112
n(14 15 50) = -0.0190
n(14 16 21) = -0.0347
n(14 16 21 47) = 0.0252
n(14 16 47 48) = 0.0178
n(14 16 47 51) = -0.0204
n(14 16 48) = 0.2360
n(14 16 48 49) = -0.0108
n(14 16 48 52) = -0.0104
n(14 16 48 53) = -0.0105
n(14 16 49 52) = 0.0131
n(14 16 52) = -0.0154
n(14 18 47) = -0.0106
n(14 18108) = -0.0147
n(14 47 48) = 0.0730
n(14 47 48 49) = 0.0631
n(14 47 48 51) = -0.0306
n(14 47 48 53) = -0.0301
n(14 47 49) = 0.0493
n(14 47 49 54) = -0.0107
n(14 47 50) = -0.0925
n(14 47 51) = -0.0548
n(14 47 51 56) = -0.0281
n(14 47 56) = -0.0216
n(14 48 49) = 0.0450
n(14 48 49 54) = -0.0107
n(14 48 52) = -0.0854
n(14 48 53) = -0.0598
n(14 48 53 60) = -0.0258
n(14 48 60) = -0.0199
n(14 49 54) = -0.0186
n(15 16 17) = 0.0149
n(15 16 21) = 0.2739
n(15 16 21 47) = 0.0117
n(15 16 47 48) = 0.0428
n(15 16 47 51) = -0.0235
n(15 16 47 52) = 0.0107
n(15 16 48 53) = -0.0232
n(15 16 50 52) = 0.0248
n(15 17 29) = -0.0135
n(15 17 31) = -0.0105
n(15 17 47) = -0.0146
n(15 19 21) = -0.0514
n(15 19 21 47) = -0.0124
n(15 19 21 52) = -0.0172
n(15 19 21 64) = -0.0356
n(15 19 21 65) = -0.0144
n(15 19 64) = -0.0109
n(15 21 47) = 0.0158
n(15 21 47 48) = 0.0102
n(15 21 47 50) = 0.0616
n(15 21 47 51) = -0.0200
n(15 21 47 52) = 0.0176
n(15 21 48) = 0.0164
n(15 21 48 52) = 0.0170
n(15 21 48 53) = -0.0132
n(15 21 50) = 0.1402
n(15 21 50 54) = -0.0152
n(15 21 52) = -0.0616
n(15 21 52 64) = -0.0180
n(15 21 64) = -0.0306
n(15 47 48) = 0.0176
n(15 47 48 53) = -0.0181
n(15 47 49) = -0.0925
n(15 47 49 50) = -0.0125
n(15 47 50) = 0.1363
n(15 47 50 54) = -0.0137
n(15 47 51) = -0.0298
n(15 47 51 55) = -0.0176
n(15 47 51 57) = 0.0110
n(15 47 55) = -0.0114
n(15 48 53) = -0.0332
n(15 49 50) = -0.0135
n(15 50 54) = -0.0376
n(16 17 31) = -0.0136
n(16 17 48) = -0.0142
n(16 19 20) = -0.0251
n(16 19 20 21) = -0.0125
n(16 19 21 50) = -0.0108
n(16 19 21 52) = 0.0104
n(16 19 21 64) = -0.0109
n(16 19 52 64) = 0.0105
n(16 21 47) = 0.0179
n(16 21 47 48) = 0.0111
n(16 21 47 50) = 0.0137
n(16 21 47 51) = -0.0135
n(16 21 48 50) = 0.0120
n(16 21 48 52) = 0.0568
n(16 21 48 53) = -0.0224
n(16 21 50) = -0.0688
n(16 21 52) = 0.1893
n(16 21 52 54) = -0.0136
n(16 47 48) = 0.0174
n(16 47 48 51) = -0.0182
n(16 47 51) = -0.0329
n(16 48 49) = -0.0926
n(16 48 49 52) = -0.0164
n(16 48 52) = 0.1654
n(16 48 52 54) = -0.0147
n(16 48 53) = -0.0392
n(16 48 53 58) = -0.0137
n(16 48 53 60) = -0.0131
n(16 49 52) = -0.0209
n(16 49 52 54) = -0.0107
n(16 50 52) = -0.0121
n(16 52 54) = -0.0467
n(16 52 54 63) = 0.0121
n(16 52 62) = -0.0136
n(17 29 30) = -0.0884
n(17 29 30 31) = -0.0222
n(17 29 30 33) = 0.0126
n(17 29 31) = 0.2509
n(17 29 31 32) = -0.0100
n(17 29 32) = -0.0644
n(17 29 32 46) = -0.0281
n(17 29 33) = 0.0192
n(17 29 33 34) = -0.0162
n(17 29 46) = -0.0219
n(17 30 31) = -0.0347
n(17 31 33 34) = -0.0221
n(17 33 34) = -0.0394
n(19 20 21) = -0.3424
n(19 20 21 27) = -0.0107
n(19 20 21 52) = -0.0147
n(19 20 21 65) = 0.0184
n(19 20 22) = -0.0100
n(19 20 22 24) = -0.0119
n(19 20 24) = -0.0246
n(19 20 26 27) = -0.0100
n(19 20 52) = -0.0418
n(19 20 52 64) = -0.0263
n(19 20 62) = -0.0120
n(19 20 62 64) = 0.0127
n(19 20 62 65) = -0.0109
n(19 20 64) = -0.0308
n(19 20 65) = -0.0396
n(19 21 27) = -0.0203
n(19 21 47) = -0.0118
n(19 21 47 50) = -0.0144
n(19 21 48 52) = -0.0151
n(19 21 49 52) = -0.0126
n(19 21 50) = -0.0197
n(19 21 50 52) = 0.0129
n(19 21 50 64) = 0.0174
n(19 21 52) = 0.0417
n(19 21 52 64) = 0.0669
n(19 21 52 65) = -0.0208
n(19 21 62 64) = -0.0183
n(19 21 64) = 0.0629
n(19 21 64 65) = -0.0641
n(19 21 64 69) = -0.0253
n(19 21 65) = -0.0578
n(19 21 69) = -0.0120
n(19 26 27) = -0.0100
n(19 47 50) = -0.0104
n(19 48 49 52) = -0.0119
n(19 48 52) = -0.0385
n(19 48 52 64) = -0.0389
n(19 49 52) = -0.0251
n(19 49 52 64) = -0.0313
n(19 50 52) = 0.0138
n(19 50 62 64) = 0.0214
n(19 52 54 64) = -0.0295
n(19 52 61 64) = 0.0109
n(19 52 62) = 0.0162
n(19 52 62 64) = 0.1349
n(19 52 62 65) = 0.0148
n(19 52 64 65) = -0.0476
n(19 52 64 68) = 0.0161
n(19 52 64 69) = 0.0769
n(19 52 65) = -0.0669
n(19 52 65 71) = -0.0111
n(19 54 62 64) = 0.0263
n(19 54 64) = -0.0299
n(19 54 64 65) = 0.0314
n(19 54 64 68) = 0.0144
n(19 54 64 69) = 0.0369
n(19 61 62) = 0.0111
n(19 61 62 65) = 0.0108
n(19 61 64) = -0.0232
n(19 61 64 65) = -0.0430
n(19 61 65) = -0.0453
n(19 61 65 66) = -0.0157
n(19 61 65 67) = -0.0123
n(19 61 65 69) = -0.0226
n(19 61 69) = -0.0114
n(19 62 64) = -0.2010
n(19 62 64 65) = -0.1243
n(19 62 64 68) = -0.0322
n(19 62 64 69) = 0.0263
n(19 62 64 70) = 0.0153
n(19 62 65 67) = -0.0101
n(19 62 65 69) = 0.0247
n(19 62 65 71) = -0.0201
n(19 62 67) = -0.0120
n(19 62 68) = -0.0102
n(19 62 69) = 0.0244
n(19 62 71) = -0.0121
n(19 64 65) = 0.0790
n(19 64 65 66) = -0.0408
n(19 64 65 68) = -0.0217
n(19 64 65 69) = -0.0660
n(19 64 65 70) = -0.0308
n(19 64 65 71) = -0.0558
n(19 64 67) = 0.0177
n(19 64 68) = -0.0177
n(19 64 68 69) = -0.0486
n(19 64 68 74) = 0.0132
n(19 64 69) = -0.0412
n(19 64 70) = 0.0129
n(19 64 71) = -0.0233
n(19 65 66) = -0.0610
n(19 65 66 71) = -0.0154
n(19 65 69) = 0.0153
n(19 65 69 71) = 0.0177
n(19 65 70) = -0.0351
n(19 65 71) = -0.0485
n(19 65 71 72) = -0.0135
n(19 68 69) = -0.0113
n(19 69 71) = 0.0158
n(19 69 72) = -0.0101
n(20 22 23 27) = 0.0253
n(20 22 24) = 0.0150
n(20 22 24 25) = -0.0201
n(20 22 24 26) = 0.0104
n(20 22 24 27) = 0.0113
n(20 22 25) = -0.0327
n(20 22 25 27) = -0.0133
n(20 22 26 27) = 0.0110
n(20 22 27) = 0.0176
n(20 23 24) = -0.0412
n(20 23 24 26) = 0.0238
n(20 23 24 75) = -0.0117
n(20 23 27) = -0.0348
n(20 23 27 75) = -0.0110
n(20 24 26) = 0.0168
n(20 24 26 28) = -0.0137
n(20 24 27) = 0.2748
n(20 24 75) = -0.0242
n(20 26 27 28) = -0.0230
n(20 26 28) = -0.0352
n(20 27 75) = -0.0234
n(20 52 64) = -0.0145
n(20 52 64 65) = 0.0187
n(20 62 64) = -0.0120
n(20 62 64 65) = -0.0140
n(20 64 65) = -0.0185
n(21 47 48 50) = 0.0103
n(21 47 48 52) = 0.0106
n(21 47 49 52) = 0.0234
n(21 47 50) = 0.0134

n(21 47 50 51) = -0.0210
n(21 47 50 52) = 0.0137
n(21 47 51) = -0.0329
n(21 47 51 52) = -0.0157
n(21 47 52) = 0.0203
n(21 48 49 50) = 0.0211
n(21 48 50) = 0.0134
n(21 48 50 53) = -0.0122
n(21 48 52 53) = -0.0233
n(21 48 52 64) = -0.0107
n(21 48 53) = -0.0332
n(21 49 50) = -0.0398
n(21 49 50 54) = -0.0143
n(21 49 52) = -0.0449
n(21 49 52 54) = -0.0125
n(21 50 52) = 0.2776
n(21 50 52 64) = 0.0203
n(21 50 54) = -0.0363
n(21 50 64) = 0.0183
n(21 52 54) = -0.0318
n(21 52 62 64) = -0.0176
n(21 52 64) = 0.0497
n(21 52 64 65) = -0.0154
n(21 52 64 69) = -0.0152
n(21 54 64 65) = 0.0117
n(21 62 64) = -0.0350
n(21 64 65) = -0.0699
n(21 64 65 69) = -0.0152
n(21 64 65 70) = 0.0120
n(21 64 69) = -0.0332
n(22 23 24) = 0.2534
n(22 23 24 26) = 0.0166
n(22 23 24 27) = 0.0316
n(22 23 25) = -0.0555
n(22 23 25 26) = -0.0309
n(22 23 25 27) = -0.0203
n(22 23 25 39) = -0.0281
n(22 23 26) = 0.0723
n(22 23 26 27) = 0.0170
n(22 23 26 28) = -0.0289
n(22 23 39) = -0.0215
n(22 23 75) = -0.0288
n(22 23 82) = -0.0105
n(22 24 25) = -0.0297
n(22 24 25 27) = -0.0234
n(22 24 25 38) = -0.0181
n(22 24 25 40) = 0.0109
n(22 24 26) = 0.0179
n(22 24 26 27) = 0.0428
n(22 24 26 28) = -0.0180
n(22 24 27 75) = -0.0113
n(22 24 38) = -0.0117
n(22 24 71) = -0.0148
n(22 24 75) = -0.0404
n(22 25 26) = -0.0744
n(22 25 26 27) = -0.0180
n(22 25 27) = -0.0323
n(22 25 38) = -0.0176
n(22 25 38169) = -0.0229
n(22 25 39) = -0.0108
n(22 25 39151) = -0.0242
n(22 25 40) = -0.0109
n(22 25 40160) = -0.0243
n(22 25151) = -0.0102
n(22 25160) = -0.0103
n(22 25169) = -0.0101
n(22 26 27) = 0.0170
n(22 26 28) = -0.0699
n(22 38169) = -0.0163
n(22 39151) = -0.0176
n(22 40160) = -0.0175
n(23 24 26 27) = 0.0320
n(23 24 26 28) = -0.0223
n(23 24 27) = 0.2546
n(23 24 27 75) = 0.0809
n(23 24 71 75) = -0.0396
n(23 24 75) = 0.1412
n(23 24 75 82) = -0.0134
n(23 26 27) = 0.2373
n(23 26 28) = -0.0539
n(23 26 28 37) = -0.0233
n(23 26 37) = -0.0171
n(23 26 75) = -0.0262
n(23 27 71 75) = -0.0378
n(23 27 75) = 0.1305
n(23 27 75 82) = -0.0116
n(23 71 75) = -0.1817
n(23 82 87) = -0.0145
n(24 26 27 28) = -0.0231
n(24 26 27 75) = -0.0111
n(24 26 28) = -0.0334
n(24 27 71) = 0.0150
n(24 27 75) = 0.1466
n(24 27 75 82) = -0.0228
n(24 66 71) = -0.0137
n(24 71 72) = -0.0108
n(24 71 75) = -0.0326
n(24 71 75 82) = 0.0209
n(24 75 82) = -0.0965
n(25 38 39) = -0.0157
n(25 38 39149) = -0.0254
n(25 38 39168) = -0.0257
n(25 38 40) = -0.0162
n(25 38 40158) = -0.0254
n(25 38 40167) = -0.0256
n(25 38149) = -0.0108
n(25 38158) = -0.0109
n(25 38167) = -0.0210
n(25 38168) = -0.0212
n(25 38169) = -0.0196
n(25 39 40) = -0.0149
n(25 39 40150) = -0.0255
n(25 39 40159) = -0.0254
n(25 39149) = -0.0220
n(25 39150) = -0.0216
n(25 39151) = -0.0202
n(25 39159) = -0.0111
n(25 39168) = -0.0112
n(25 40150) = -0.0112
n(25 40158) = -0.0216
n(25 40159) = -0.0218
n(25 40160) = -0.0196
n(25 40167) = -0.0114
n(26 27 28) = -0.0404
n(26 27 28 35) = -0.0104
n(26 27 28 37) = -0.0163
n(26 27 37) = -0.0116
n(26 27 65) = -0.0134
n(26 27 71) = -0.0142
n(26 27 75) = -0.0381
n(26 28 35) = -0.0126
n(26 28 35166) = -0.0231
n(26 28 36) = -0.0191
n(26 28 36141) = -0.0234
n(26 28 37) = -0.0135
n(26 28 37157) = -0.0232
n(26 28141) = -0.0102
n(26 28157) = -0.0105
n(26 28166) = -0.0100
n(26 35166) = -0.0169
n(26 36141) = -0.0164
n(26 37157) = -0.0153
n(27 62 65) = -0.0124
n(27 65 71) = 0.0114
n(27 71 72) = -0.0133
n(27 71 75) = -0.0509
n(27 71 75 82) = 0.0231
n(27 75 82) = -0.0891
n(28 35 36) = -0.0149
n(28 35 36140) = -0.0255
n(28 35 36164) = -0.0254
n(28 35 37) = -0.0134
n(28 35 37155) = -0.0256
n(28 35 37165) = -0.0260
n(28 35140) = -0.0110
n(28 35155) = -0.0106
n(28 35164) = -0.0216
n(28 35165) = -0.0209
n(28 35166) = -0.0198
n(28 36 37) = -0.0157
n(28 36 37142) = -0.0258
n(28 36 37156) = -0.0260
n(28 36140) = -0.0221
n(28 36141) = -0.0197
n(28 36142) = -0.0220
n(28 36156) = -0.0110
n(28 36164) = -0.0111
n(28 37142) = -0.0113
n(28 37155) = -0.0231
n(28 37156) = -0.0220
n(28 37157) = -0.0200
n(28 37165) = -0.0114
n(29 30 31) = 0.2088
n(29 30 31 33) = 0.0486
n(29 30 32) = -0.0290
n(29 30 32 33) = -0.0219
n(29 30 32 45) = -0.0255
n(29 30 33) = 0.0201
n(29 30 33 34) = -0.0274
n(29 30 45) = -0.0178
n(29 31 32) = -0.0153
n(29 31 32 33) = -0.0233
n(29 31 32 44) = 0.0126
n(29 31 32 45) = -0.0152
n(29 31 32 46) = -0.0123
n(29 31 33) = 0.0198
n(29 31 33 34) = -0.0276
n(29 32 33) = -0.0405
n(29 32 44) = -0.0164
n(29 32 44128) = -0.0240
n(29 32 45) = -0.0123
n(29 32 45 94) = -0.0237
n(29 32 46118) = -0.0231
n(29 32118) = -0.0105
n(29 32128) = -0.0103
n(29 33 34) = -0.0500
n(29 44128) = -0.0173
n(29 45 94) = -0.0177
n(29 46118) = -0.0157
n(30 31 33) = 0.1643
n(30 32 45) = -0.0115
n(30 33 34) = -0.0438
n(30 33 34 42) = -0.0261
n(30 33 42) = -0.0196
n(31 32 33) = -0.0103
n(31 33 34) = -0.0509
n(31 33 34 41) = -0.0256
n(31 33 41) = -0.0193
n(31 44128) = -0.0118
n(32 44 45) = -0.0143
n(32 44 45 92) = -0.0256
n(32 44 45130) = -0.0253
n(32 44 46) = -0.0159
n(32 44 46117) = -0.0257
n(32 44 46129) = -0.0259
n(32 44 92) = -0.0107
n(32 44117) = -0.0115
n(32 44128) = -0.0206
n(32 44129) = -0.0219
n(32 44130) = -0.0227
n(32 45 46) = -0.0144
n(32 45 46 93) = -0.0257
n(32 45 46116) = -0.0254
n(32 45 92) = -0.0224
n(32 45 93) = -0.0211
n(32 45 94) = -0.0208
n(32 45116) = -0.0108
n(32 45130) = -0.0105
n(32 46 93) = -0.0113
n(32 46116) = -0.0215
n(32 46117) = -0.0210
n(32 46118) = -0.0192
n(32 46129) = -0.0115
n(33 34 41) = -0.0131
n(33 34 41101) = -0.0240
n(33 34 42) = -0.0103
n(33 34 42137) = -0.0235
n(33 34 43) = -0.0143
n(33 34 43148) = -0.0228
n(33 34101) = -0.0101
n(33 34137) = -0.0101
n(33 34148) = -0.0105
n(33 41101) = -0.0174
n(33 42137) = -0.0170
n(33 43148) = -0.0156
n(34 41 42) = -0.0155
n(34 41 42103) = -0.0254
n(34 41 42139) = -0.0254
n(34 41 43) = -0.0174
n(34 41 43102) = -0.0255
n(34 41 43147) = -0.0256
n(34 41101) = -0.0199
n(34 41102) = -0.0223
n(34 41103) = -0.0218
n(34 41139) = -0.0110
n(34 41147) = -0.0115
n(34 42 43) = -0.0182
n(34 42 43138) = -0.0255
n(34 42 43146) = -0.0254
n(34 42103) = -0.0110
n(34 42137) = -0.0193
n(34 42138) = -0.0209
n(34 42139) = -0.0216
n(34 42146) = -0.0115
n(34 43102) = -0.0111
n(34 43138) = -0.0116
n(34 43146) = -0.0206
n(34 43147) = -0.0206
n(34 43148) = -0.0183
n(35 36140) = -0.0163
n(35 36164) = -0.0160
n(35 37155) = -0.0169
n(35 37165) = -0.0165
n(36 37142) = -0.0161
n(36 37156) = -0.0167
n(38 39149) = -0.0162
n(38 39168) = -0.0161
n(38 40158) = -0.0160
n(38 40167) = -0.0158
n(39 40150) = -0.0161
n(39 40159) = -0.0160
n(41 42103) = -0.0161
n(41 42139) = -0.0159
n(41 43102) = -0.0158
n(41 43147) = -0.0157
n(42 43138) = -0.0154
n(42 43146) = -0.0153
n(44 45 92) = -0.0165
n(44 45130) = -0.0166
n(44 46117) = -0.0159
n(44 46129) = -0.0160
n(45 46 93) = -0.0161
n(45 46116) = -0.0162
n(47 48 49) = 0.0746
n(47 48 49 50) = 0.0152
n(47 48 49 51) = -0.0320
n(47 48 49 52) = 0.0214
n(47 48 49 53) = -0.0301
n(47 48 50) = 0.0153
n(47 48 50 52) = 0.0427
n(47 48 50 53) = -0.0168
n(47 48 51) = -0.0743
n(47 48 51 52) = -0.0208
n(47 48 52) = 0.0206
n(47 48 53) = -0.0724
n(47 49 50) = 0.2168
n(47 49 50 52) = 0.0425
n(47 49 51) = -0.0602
n(47 49 51 52) = -0.0225
n(47 49 51 57) = -0.0289
n(47 49 52) = 0.0193
n(47 49 54) = -0.0268
n(47 49 57) = -0.0227
n(47 50 51) = -0.0319
n(47 50 51 52) = -0.0257
n(47 50 51 55) = -0.0208
n(47 50 51 56) = 0.0104
n(47 50 52) = 0.0159
n(47 50 54) = -0.0267
n(47 50 55) = -0.0139
n(47 50 64) = -0.0138
n(47 51 52) = -0.0403
n(47 51 55) = -0.0176
n(47 51 55 84) = -0.0229
n(47 51 56) = -0.0105
n(47 51 56119) = -0.0242
n(47 51 57) = -0.0111
n(47 51 57163) = -0.0243
n(47 51 84) = -0.0101
n(47 51119) = -0.0102
n(47 51163) = -0.0104
n(47 55 84) = -0.0163
n(47 56119) = -0.0176
n(47 57163) = -0.0176
n(48 49 50 52) = 0.0236
n(48 49 50 53) = -0.0175
n(48 49 52) = 0.2335
n(48 49 52 53) = -0.0105
n(48 49 52 64) = -0.0155
n(48 49 53) = -0.0503
n(48 49 53 59) = -0.0269
n(48 49 54) = -0.0317
n(48 49 59) = -0.0190
n(48 50 52 53) = -0.0231
n(48 50 53) = -0.0287
n(48 52 53) = -0.0429
n(48 52 53 58) = -0.0251
n(48 52 54) = -0.0290
n(48 52 58) = -0.0174
n(48 52 62) = -0.0244
n(48 52 62 64) = -0.0132
n(48 52 64) = -0.0656
n(48 52 65) = -0.0169
n(48 53 58) = -0.0154
n(48 53 58127) = -0.0229
n(48 53 59) = -0.0174
n(48 53 59131) = -0.0236
n(48 53 60) = -0.0113
n(48 53 60115) = -0.0233
n(48 53115) = -0.0102
n(48 53127) = -0.0102
n(48 53131) = -0.0102
n(48 58127) = -0.0162
n(48 59131) = -0.0167
n(48 60115) = -0.0161
n(49 50 52) = 0.2260
n(49 50 52 54) = 0.0900
n(49 50 54) = 0.1474
n(49 50 54 62) = -0.0170
n(49 50 54 63) = -0.0102
n(49 52 54) = 0.1656
n(49 52 54 62) = -0.0200
n(49 52 54 63) = -0.0251
n(49 52 64) = -0.0318
n(49 53 59) = -0.0108
n(49 54 62) = -0.1299
n(49 54 63) = -0.0447
n(50 52 54) = 0.1619
n(50 52 54 63) = -0.0285
n(50 52 54 64) = 0.0122
n(50 52 62) = 0.0117
n(50 52 64) = 0.0137
n(50 54 62) = -0.0378
n(50 54 63) = -0.0758
n(50 54 64) = 0.0152
n(50 54 64 65) = -0.0103
n(50 62 68) = -0.0126
n(50 64 65) = -0.0191
n(51 55 56) = -0.0156
n(51 55 56 85) = -0.0257
n(51 55 56121) = -0.0254
n(51 55 57) = -0.0163
n(51 55 57 83) = -0.0256
n(51 55 57162) = -0.0254
n(51 55 83) = -0.0211
n(51 55 84) = -0.0196
n(51 55 85) = -0.0211
n(51 55121) = -0.0108
n(51 55162) = -0.0109
n(51 56 57) = -0.0150
n(51 56 57120) = -0.0255
n(51 56 57161) = -0.0254
n(51 56 85) = -0.0112
n(51 56119) = -0.0203
n(51 56120) = -0.0216
n(51 56121) = -0.0221
n(51 56161) = -0.0112
n(51 57 83) = -0.0113
n(51 57120) = -0.0112
n(51 57161) = -0.0217
n(51 57162) = -0.0217
n(51 57163) = -0.0195
n(52 53 58) = -0.0108
n(52 54 62) = 0.0108
n(52 54 62 64) = 0.0136
n(52 54 63) = -0.1358
n(52 54 64) = 0.0149
n(52 62 64 65) = 0.0258
n(52 62 64 67) = -0.0114
n(52 62 64 68) = -0.0146
n(52 62 65) = 0.0136
n(52 62 67) = -0.0219
n(52 62 68) = -0.0166
n(52 64 65) = -0.1309
n(52 64 65 70) = 0.0123
n(52 64 65 71) = -0.0120
n(52 64 69) = -0.0742
n(52 65 71) = -0.0101
n(53 58 59) = -0.0159
n(53 58 59126) = -0.0255
n(53 58 59132) = -0.0254
n(53 58 60) = -0.0149
n(53 58 60113) = -0.0255
n(53 58 60125) = -0.0259
n(53 58113) = -0.0110
n(53 58125) = -0.0207
n(53 58126) = -0.0213
n(53 58127) = -0.0196
n(53 58132) = -0.0111
n(53 59 60) = -0.0145
n(53 59 60114) = -0.0258
n(53 59 60133) = -0.0258
n(53 59114) = -0.0108
n(53 59126) = -0.0112
n(53 59131) = -0.0199
n(53 59132) = -0.0224
n(53 59133) = -0.0226
n(53 60113) = -0.0222
n(53 60114) = -0.0224
n(53 60115) = -0.0205
n(53 60125) = -0.0115
n(53 60133) = -0.0109
n(54 62 64) = 0.0149
n(54 62 64 65) = -0.0136
n(54 62 64 68) = -0.0122
n(54 62 64 69) = -0.0161
n(54 62 67) = -0.0195
n(54 62 67 68) = -0.0110
n(54 62 68) = -0.0443
n(54 62 69) = -0.0139
n(54 63143) = -0.0137
n(54 63144) = -0.0101
n(54 63145) = -0.0119
n(54 64 65) = -0.0340
n(54 64 69) = -0.0172
n(55 56 85) = -0.0161
n(55 56121) = -0.0162
n(55 57 83) = -0.0158
n(55 57162) = -0.0160
n(56 57120) = -0.0162
n(56 57161) = -0.0159
n(58 59126) = -0.0159
n(58 59132) = -0.0158
n(58 60113) = -0.0163
n(58 60125) = -0.0161
n(59 60114) = -0.0167
n(59 60133) = -0.0166
n(61 62 64) = 0.0101
n(61 62 64 65) = 0.0308
n(61 62 65) = 0.2766
n(61 62 65 66) = 0.0243
n(61 62 65 69) = -0.0142
n(61 62 66 67) = 0.0328
n(61 62 66 68) = 0.0300
n(61 62 66 73) = -0.0321

n(61 62 67) = 0.2677
n(61 62 67 68) = 0.0132
n(61 62 68) = -0.0498
n(61 62 68 74) = -0.0241
n(61 62 69) = -0.0421
n(61 62 69 71) = 0.0124
n(61 64 65) = -0.0212
n(61 64 65 69) = -0.0243
n(61 64 69) = -0.0134
n(61 65 66) = 0.1115
n(61 65 66 71) = 0.0409
n(61 65 66 73) = -0.0228
n(61 65 67) = -0.0442
n(61 65 67 68) = 0.0183
n(61 65 68) = 0.0109
n(61 65 68 74) = -0.0115
n(61 65 69) = -0.0648
n(61 65 69 71) = -0.0150
n(61 65 71) = -0.0191
n(61 66 67) = 0.2270
n(61 66 67 71) = -0.0126
n(61 66 67 72) = -0.0281
n(61 66 68) = 0.0132
n(61 66 68 74) = -0.0124
n(61 66 71) = -0.0228
n(61 66 71 72) = -0.0164
n(61 66 72) = -0.0976
n(61 66 73) = -0.0553
n(61 66 73 76) = -0.0242
n(61 66 76) = -0.0175
n(61 67 68) = -0.0260
n(61 67 68 72) = -0.0113
n(61 67 68 74) = -0.0169
n(61 67 72) = -0.0400
n(61 68 74) = -0.0249
n(62 63 65) = -0.0108

n(62 63143) = -0.0149
n(62 64 65) = 0.0637
n(62 64 65 69) = 0.0556
n(62 64 65 71) = -0.0265
n(62 64 67) = -0.0324
n(62 64 68) = -0.0136
n(62 64 68 69) = 0.0190
n(62 64 69) = 0.0447
n(62 64 70) = -0.0181
n(62 64 71) = -0.0132
n(62 65 66) = 0.0241
n(62 65 66 67) = 0.0416
n(62 65 66 68) = 0.0133
n(62 65 66 73) = -0.0245
n(62 65 67) = -0.0225
n(62 65 68) = 0.0129
n(62 65 68 69) = 0.0610
n(62 65 68 71) = 0.0229
n(62 65 68 74) = -0.0294
n(62 65 69) = 0.2042
n(62 65 71) = -0.0495
n(62 65 71 75) = -0.0112
n(62 65 75) = -0.0101
n(62 66 67) = 0.0150
n(62 66 67 68) = 0.0135
n(62 66 67 73) = -0.0209
n(62 66 68) = 0.0222
n(62 66 68 72) = 0.0175
n(62 66 68 73) = -0.0183
n(62 66 69) = 0.0108
n(62 66 69 71) = 0.0155
n(62 66 73) = -0.0483
n(62 67 68) = 0.2230
n(62 67 68 69) = -0.0135
n(62 67 68 72) = -0.0136
n(62 67 68 74) = -0.0102

n(62 67 69) = -0.0149
n(62 67 69 72) = 0.0180
n(62 67 72) = -0.0158
n(62 68 69) = 0.1681
n(62 68 72) = -0.0834
n(62 68 74) = -0.0671
n(62 68 74 81) = -0.0215
n(62 68 81) = -0.0160
n(62 69 71) = -0.0193
n(64 65 66) = -0.0379
n(64 65 69) = 0.0625
n(64 65 69 71) = 0.0236
n(64 65 70) = -0.0423
n(64 65 71) = -0.0441
n(64 69 72) = -0.0176
n(64 70110) = -0.0123
n(64 70112) = -0.0123
n(65 66 68 69) = 0.0147
n(65 66 69) = 0.0126
n(65 66 69 71) = 0.0194
n(65 66 69 72) = 0.0332
n(65 66 69 73) = -0.0183
n(65 66 71) = 0.1314
n(65 66 71 73) = -0.0266
n(65 66 72) = -0.0171
n(65 66 73) = -0.0716
n(65 68 69) = 0.0222
n(65 68 69 71) = 0.0136
n(65 68 69 74) = -0.0247
n(65 68 71) = 0.0185
n(65 68 71 72) = 0.0170
n(65 68 71 74) = -0.0153
n(65 68 74) = -0.0430
n(65 69 71) = 0.2792
n(65 69 72) = -0.0105
n(65 71 72) = -0.0519

n(66 67 68) = 0.0211
n(66 67 68 69) = 0.0116
n(66 67 68 71) = 0.0120
n(66 67 68 72) = 0.0489
n(66 67 68 73) = -0.0220
n(66 67 68 74) = -0.0276
n(66 67 71) = -0.0881
n(66 67 71 72) = -0.0223
n(66 67 72) = 0.2069
n(66 67 73) = -0.0309
n(66 67 73 77) = -0.0255
n(66 67 77) = -0.0179
n(66 68 69) = 0.0199
n(66 68 69 71) = 0.0306
n(66 68 69 72) = 0.0121
n(66 68 69 73) = -0.0151
n(66 68 71) = 0.0192
n(66 68 71 74) = -0.0162
n(66 68 72) = 0.0206
n(66 68 72 73) = -0.0234
n(66 68 72 74) = -0.0280
n(66 68 73) = -0.0410
n(66 68 74) = -0.0510
n(66 69 71) = -0.0269
n(66 69 71 72) = 0.0155
n(66 69 71 73) = -0.0293
n(66 69 72 73) = -0.0132
n(66 69 73) = -0.0332
n(66 71 72) = 0.2501
n(66 71 72 75) = -0.0134
n(66 71 73) = -0.0623
n(66 71 73 78) = -0.0281
n(66 71 75) = -0.0283
n(66 71 78) = -0.0218
n(66 72 73) = -0.0140
n(66 72 73 76) = 0.0128

n(66 72 73 77) = -0.0151
n(66 72 73 78) = -0.0125
n(66 73 76) = -0.0165
n(66 73 76124) = -0.0240
n(66 73 77) = -0.0125
n(66 73 77104) = -0.0237
n(66 73 78172) = -0.0231
n(66 73124) = -0.0103
n(66 73172) = -0.0105
n(66 76124) = -0.0173
n(66 77104) = -0.0177
n(66 78172) = -0.0157
n(67 68 69) = -0.0911
n(67 68 69 72) = -0.0120
n(67 68 72) = 0.1610
n(67 68 74) = -0.0445
n(67 68 74 80) = -0.0268
n(67 68 80) = -0.0201
n(67 69 72) = -0.0120
n(67 71 72) = -0.0348
n(67 73 77) = -0.0113
n(67 74 80) = -0.0101
n(68 69 71 72) = 0.0496
n(68 69 71 74) = -0.0295
n(68 69 71 75) = -0.0110
n(68 69 72) = 0.1693
n(68 69 74) = -0.0625
n(68 69 74 79) = -0.0124
n(68 69 74 81) = -0.0172
n(68 69 81) = -0.0133
n(68 71 72 74) = -0.0220
n(68 71 74) = -0.0393
n(68 71 75) = -0.0102
